# Distinct pretreatment innate immune landscape and posttreatment T cell responses underlie immunotherapy-induced colitis

**DOI:** 10.1172/jci.insight.157839

**Published:** 2022-11-08

**Authors:** Kazi J. Nahar, Felix Marsh-Wakefield, Robert V. Rawson, Tuba N. Gide, Angela L. Ferguson, Ruth Allen, Camelia Quek, Ines Pires da Silva, Stephen Tattersal, Christopher J. Kiely, Neomal Sandanayake, Matteo S. Carlino, Geoff McCaughan, James S. Wilmott, Richard A. Scolyer, Georgina V. Long, Alexander M. Menzies, Umaimainthan Palendira

**Affiliations:** 1 Melanoma Institute Australia,; 2 Faculty of Medicine and Health,; 3 Charles Perkins Centre, and; 4 Centenary Institute, The University of Sydney, Sydney, New South Wales, Australia.; 5 Department of Tissue Pathology and Diagnostic Oncology, Royal Prince Alfred Hospital and NSW Health Pathology, Sydney, New South Wales, Australia.; 6 Royal North Shore Hospital, Sydney, New South Wales Australia.; 7 Crown Princess Mary Cancer Centre and Westmead Hospitals, New South Wales, Australia.; 8 Mater Hospital, North Sydney, New South Wales, Australia.

**Keywords:** Immunology, Oncology, Autoimmune diseases, Cancer immunotherapy, Melanoma

## Abstract

Immune-related adverse events represent a major hurdle to the success of immunotherapy. The immunological mechanisms underlying their development and relation to antitumor responses are poorly understood. By examining both systemic and tissue-specific immune changes induced by combination anti–CTLA-4 and anti–PD-1 immunotherapy, we found distinct repertoire changes in patients who developed moderate-severe colitis, irrespective of their antitumor response to therapy. The proportion of circulating monocytes were significantly increased at baseline in patients who subsequently developed colitis compared with patients who did not develop colitis, and biopsies from patients with colitis showed monocytic infiltration of both endoscopically and histopathologically normal and inflamed regions of colon. The magnitude of systemic expansion of T cells following commencement of immunotherapy was also greater in patients who developed colitis. Importantly, we show expansion of specific T cell subsets within inflamed regions of the colon, including tissue-resident memory CD8^+^ T cells and Th1 CD4^+^ T cells in patients who developed colitis. Our data also suggest that CD8^+^ T cell expansion was locally induced, while Th1 cell expansion was systemic. Together, our data show that exaggerated innate and T cell responses to combination immunotherapy synergize to propel colitis in susceptible patients.

## Introduction

Antibodies against the immune checkpoints CTLA-4, PD-1, and its ligand PD-L1 are now important treatments across oncology, and new drugs targeting other checkpoints are in development. The combination of ipilimumab (IPI; anti–CTLA-4) and nivolumab (anti–PD-1) demonstrates superior response and survival in melanoma compared with single-agent nivolumab or IPI and, therefore, has become the preferred first-line regimen for many patients with advanced disease (1). Apart from melanoma, combination immune checkpoint inhibitors are now approved by the U.S. Food and Drug Administration (FDA) for treatment of several cancers, including advanced non–small cell lung cancer, advanced renal cell cancer, hepatocellular cancer, malignant mesothelioma, and metastatic colorectal cancer with mismatch repair–deficient/microsatellite instability-high aberrations (2–6).

While immunotherapy intends to restore antitumor immunity, it also often leads to immune activation in normal host tissue, resulting in immune-related adverse events (irAEs) (7). Such irAEs are frequent, can be severe, and result in morbidity and rarely death. While they may mimic idiopathic autoimmune diseases, their clinical course, management, and outcomes appear distinct. The incidence of irAEs is high, ranging from 54% to 76% depending on the treatment, and is highest in patients treated with combination anti–CTLA-4 and anti–PD-1 therapy, where at least one-third of the patients develop moderate-severe (grade 3 or 4, according to the Common Terminology Criteria for Adverse Events, CTCAE v.5.0) toxicity (8, 9). Although irAEs tend to develop early, between 4 and 6 weeks from the start of immunotherapy, some cases have developed 3 years later (10). IrAEs can affect any organ of the body but most commonly affect the gastrointestinal tract, endocrine glands, skin, and liver. Notably, irAEs involving the gastrointestinal tract were the most common cause of toxicity, leading to treatment discontinuation in clinical trials, occurring up to 13.6% with combination therapy (11).

Mechanisms underlying the development of irAEs are not well understood, and whether they are similar to classical autoimmune diseases is unclear (12). Whether antitumor responses play a role in the development of irAEs and whether irAEs could be treated without compromising tumor responses are poorly understood. Moreover, early recognition of patients prone to develop severe irAEs could allow early intervention and minimize discontinuation rate. Early diversification of T cell repertoire and increased T cell receptor clonotypes were associated with irAEs in patients with metastatic prostate cancer treated with anti–CTLA-4 therapy (13). In a similar study, clonal expansion of CD8^+^ T cells was associated with moderate-severe toxicity (14). Gene expression profiling in IPI-treated patients suggested high expression of CD177, a neutrophil marker, to be predictive of colitis development (15). High levels of IL-17 at baseline have been associated with colitis in patients with metastatic melanoma treated with IPI (16). A recent study performed single-cell RNA-Seq of colon biopsies in combination immunotherapy–treated patients and demonstrated a potential role for CD8^+^ tissue-resident memory (Trm) T cells in colitis (17). Most studies thus far have focused on either the systemic immune responses or local responses independently and, therefore, have not determined the full impact of immune perturbations associated with the development of irAEs.

In this study, we have utilized high-dimensional mass cytometry to interrogate circulating immune cells and the systemic immune responses in patients with melanoma who did and did not develop colitis on-treatment with combination therapy of anti–CTLA-4 and anti–PD-1 (18). Paired colon biopsies from patients who developed colitis (including areas with and without clinically apparent colitis) were investigated by multiplex IHC (mIHC) to map the immune landscape of the end-organ. We show a complex interaction between multiple innate and adaptive immune cells that likely drive the immunopathology of moderate-severe colitis. In particular, we show that the innate immune repertoire of patients prior to immunotherapy could make patients susceptible to colitis and that immunotherapy-induced changes associated with colitis are independent of antitumor responses.

## Results

### Study design.

Thirty-seven patients with metastatic melanoma treated with combination immunotherapy (IPI and either nivolumab or pembrolizumab) were selected for this study; 19 who had developed moderate-severe (≥grade 2–4) colitis on-treatment and 18 who did not develop any signs of colitis or other significant toxicity (no-colitis; ≤ grade 2 rash and thyroiditis were permitted). Mass cytometry analysis (by CyTOF) of peripheral blood mononuclear cells (PBMC) taken from these patients at baseline (T_0_, pretreatment) and at the time of colitis (T_1_, or a matched time point for the no-colitis group) was performed to define systemic immune composition and therapy-induced changes.

Twenty-six colon biopsy specimens were taken from patients who developed moderate-severe colitis, and they were examined by mIHC. A subset of patients from whom colon biopsies were taken (7 of 26) had additional biopsies taken from regions deemed endoscopically and histopathologically normal for mIHC staining (Table 1 and Supplemental Table 1; supplemental material available online with this article; https://doi.org/10.1172/jci.insight.157839DS1).

### Circulating innate immune profile at baseline is associated with immunotherapy-induced colitis.

**Figure 1:**
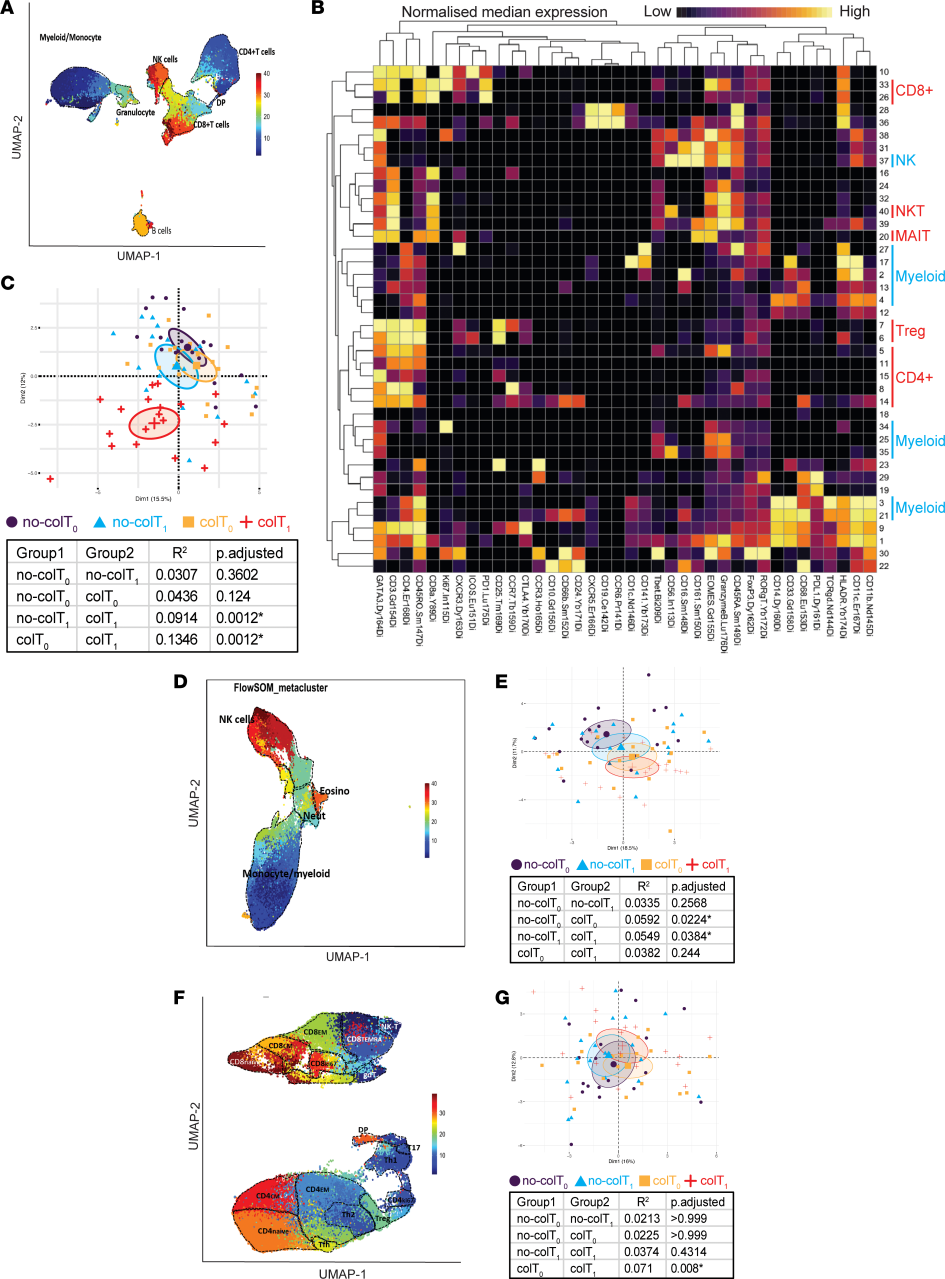
Patients who develop colitis have a distinct innate immune repertoire prior to treatment. CyTOF analysis followed by FlowSOM clustering was performed on PBMCs to determine the immune repertoire of patients. (**A**) UMAP plot visualizing major cell populations as annotated from all samples (*n* = 37) on both time points and the colors represent the 40 metaclusters generated. (**B**) Heatmap showing relative median signal intensity of markers (columns) for each metacluster (rows). Myeloid (blue) and T cell (red) subsets are annotated. (**C**) A PCA plot was generated on the data of patients (*n* = 37) with metacluster levels (as proportion of PBMC) as variables. A PERMANOVA was performed on the scaled Euclidean distance of all patients. Patients were grouped based on colitis before and after treatment. Myeloid cells and T cells were then gated on manually, followed by FlowSOM clustering. (**D** and **F**) UMAP of myeloid cells (**D**) and T cells (**F**) from all samples (*n* = 37) on both time points with annotated subsets. (**E** and **G**) PCA plot and PERMANOVA of myeloid cells (**E**) and T cells (**G**). Brown-Forsythe and Welch ANOVA with Dunnett T3 multiple-comparison test (unpaired groups) or mixed-effects analysis with Šídák’s multiple-comparison test (paired time points) were used. Median shown. *P ≤ 0.05. no‑colT_0_, no-colitis baseline; no‑colT_1_, no-colitis treatment; colT_0_, colitis baseline; colT_1_, colitis treatment.

**Figure 2:**
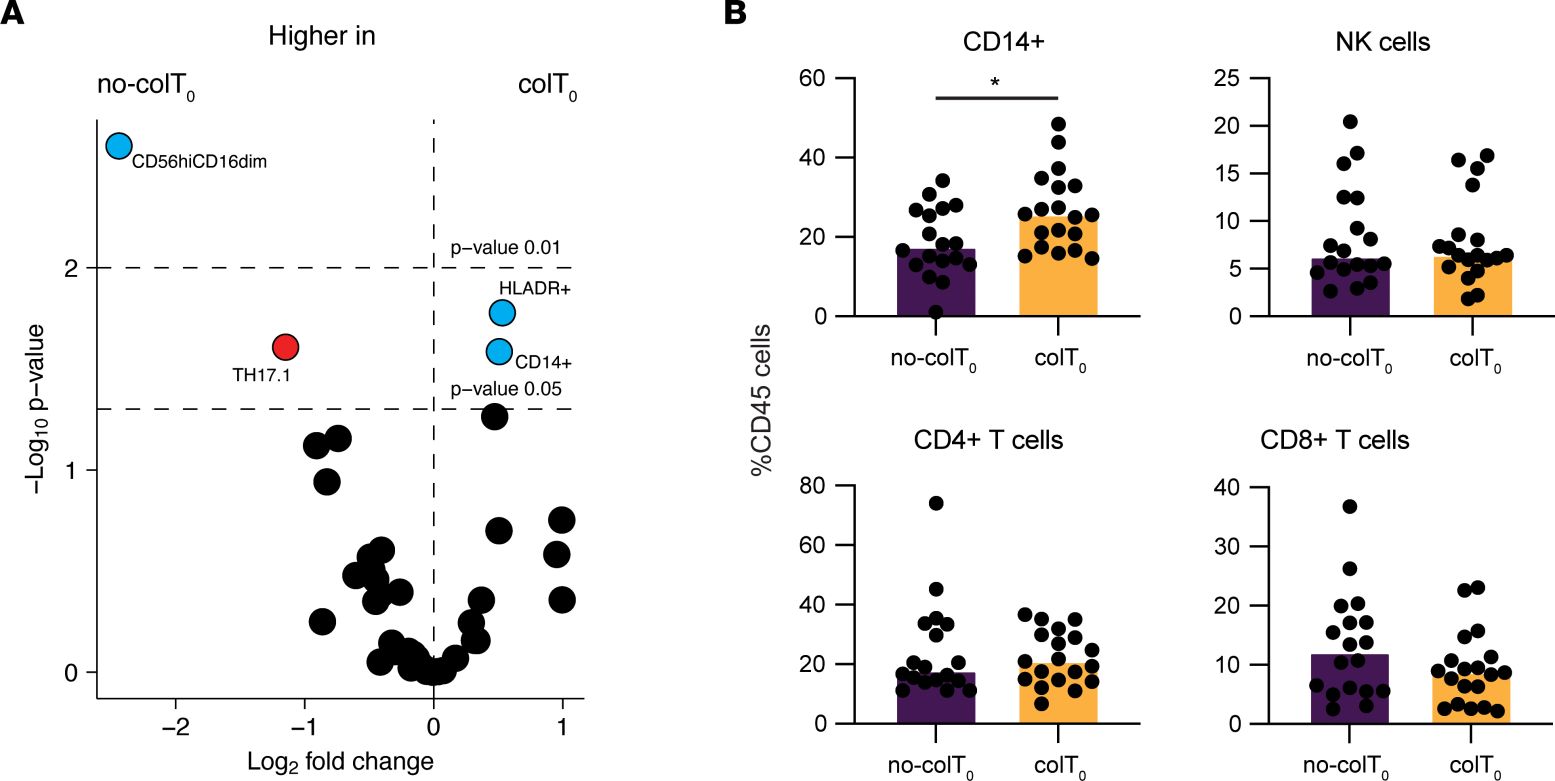
Difference in circulating monocytes at baseline is associated with the development of severe colitis. Manual gating was performed on major myeloid cell and T cell subsets to identify immune populations that were associated with the development of immunotherapy-induced colitis. (**A**) Volcano plot summarizes the changes between baselines of colitis (col) and no-colitis (no-col) patients. Subsets with *P* ≤ 0.05 are annotated. Blue circles are myeloid cell subsets; red circles are T cells. (**B**) Proportions of cells expressing the markers were determined by manual gating between patients who developed colitis and those who did not. Brown-Forsythe and Welch ANOVA with Dunnett T3 multiple-comparison test (unpaired groups) or mixed-effects analysis with Šídák’s multiple-comparison test (paired time points) were used. Median shown. *P ≤ 0.05. no‑colT_0_, no-colitis baseline; no‑colT_1_, no-colitis treatment; colT_0_, colitis baseline; colT_1_, colitis treatment.

Only a subset of patients receiving combination therapy develop severe colitis. In order to determine whether the immune profile of patients prior to the start of immunotherapy had any impact on the development of colitis, we performed multiparameter analysis by CyTOF on PBMC taken before and during treatment. Samples were divided into 4 groups: baseline no-colitis (no-colT_0_), treatment no-colitis (no-colT_1_), baseline colitis (colT_0_), and treatment colitis (colT_1_). Unsupervised FlowSOM clustering of the data generated 40 metaclusters covering both innate and adaptive immune populations (Figure 1, A and B). To determine whether the overall immune cell profiles of individual patients were different between the 4 groups, principal component analysis (PCA) and permutational multivariate ANOVA (PERMANOVA) were used. These revealed that patients who developed colitis had the most significantly different profile, and this was more apparent during treatment (Figure 1C). In order to determine whether any differences at baseline were associated with colitis, we performed deeper immune phenotyping of the immune repertoire by separating T cells from other immune populations — namely, innate immune populations. Unsupervised FlowSOM clustering was used to generate a further 40 metaclusters for both T cells and innate immune cells. The PCA and PERMANOVA of these clusters revealed that the baseline innate immune cell makeup was significantly different between those who developed colitis and those who did not develop any symptoms of colitis (Figure 1, D and E). Interestingly, a similar analysis of T cell populations showed no significant differences at baseline (Figure 1, F and G). In order to validate these findings, we used manual gating to identify 56 immune populations from the entire immune profile and examined the magnitude of the differences that were statistically significant. This showed that multiple innate populations were indeed significantly different at baseline between patients who developed colitis when compared with those who did not (Figure 2 and Supplemental Figure 1E). Importantly, total CD14^+^ monocytes (Figure 2B) were among those that were different at baseline. There was also a difference in a subset of NK cells, with CD56^hi^CD16^dim^ NK cells significantly higher in patients who did not develop colitis (Figure 2B and Supplemental Figure 1E). There were no significant differences in total CD3, CD4, and CD8 T cells, nor were there significant differences in any of their major subsets (Figure 2, A and B, and Supplemental Figure 1F). Together, these data demonstrate that the innate immune cell composition, particularly monocyte subsets, are potentially critical for the development of immunotherapy-induced colitis.

### A multitude of systemic changes to the immune profile are associated with combination therapy.

**Figure 3:**
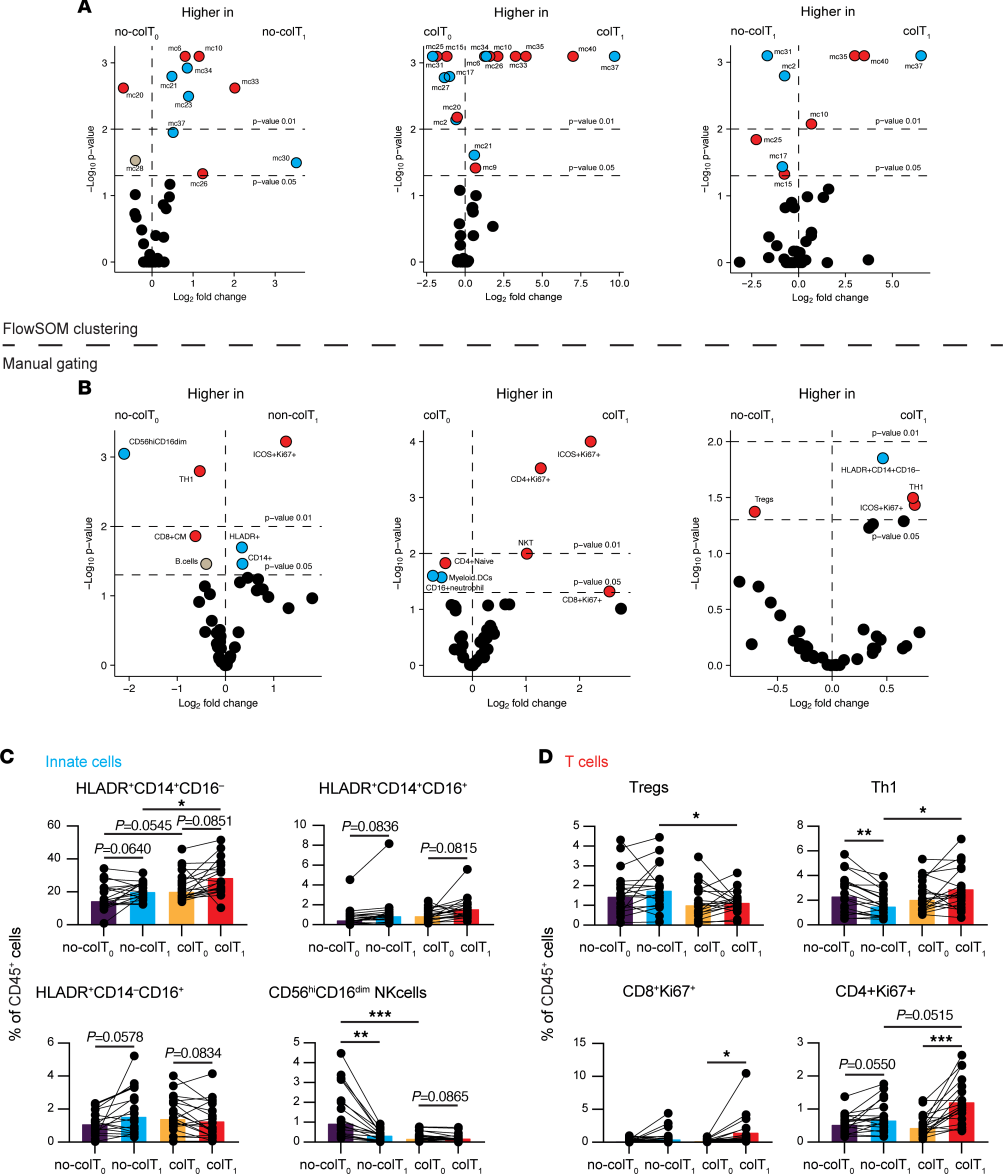
T cell subsets are altered after treatment, irrespective of colitis. (**A**) Volcano plots representing metaclusters that are changed between groups. Metaclusters with *P* ≤ 0.05 are annotated. Blue circles are metaclusters phenotypically resembling innate cells, red circles are T cells, and gray circles are other cell subsets. (**B**) Volcano plots representing differences between colitis (col) and no-colitis (no-col) groups. Blue circles are innate cell subsets, red circles are T cell subsets, and gray circles are other cell subsets. Subsets with *P* ≤ 0.05 are annotated. (**C** and **D**) Selected innate (**C**) and T cell (**D**) subsets are shown. Brown-Forsythe and Welch ANOVA with Dunnett T3 multiple-comparison test (unpaired groups) or mixed-effects analysis with Šídák’s multiple-comparison test (paired time points) were used. Median shown. *P ≤ 0.05, **P ≤ 0.01, ***P ≤ 0.001, ****P ≤ 0.0001. *no‑colT_0_*, no-colitis baseline; no‑colT_1_, no-colitis treatment; colT_0_, colitis baseline; colT_1_, colitis treatment; mc, metacluster.

We next sought to determine what changes were associated with combination immunotherapy and whether any of those changes were specific to patients who developed colitis. To this end, we compared the immune profile at baseline against the immune profile on-treatment (at the development of colitis or a corresponding time point for those without colitis). When we compared the global systemic changes between baseline and on-treatment using both unsupervised FlowSOM clustering (Figure 3A) and manual gating strategies (Figure 3B), immunotherapy-induced changes were apparent in both colitis and no-colitis cohorts. These included changes to innate populations, as well as T cells, although changes to T cells were more dominant (Figure 3, A and B). Among the populations changed during treatment were the CD14^+^ monocytes. We saw increases in the proportions of HLA-DR^+^CD14^+^CD16^–^ monocytes and HLA-DR^+^CD14^+^CD16^+^ monocytes, although the values did not reach significance. However, none of these changes were specific for patients who developed colitis (Figure 3C), suggesting that these are common for all patients undergoing combination treatment. Interestingly, CD56^hi^CD16^dim^ NK cell proportions were significantly reduced between baseline and on-treatment. This reduction was significant only in those who developed colitis — not those who did not (Figure 3C). Therapy-associated changes to T cells included an expansion of ICOS^+^Ki67^+^CD4^+^ T cells (Supplemental Figure 1F) and Ki67^+^ proliferative CD4^+^ and CD8^+^ T cells (Figure 3D and Supplemental Figure 1F). A significant expansion of proliferative CD8^+^ T cells was only present in those who developed colitis, suggesting that it could be involved in the development of colitis (Figure 3D). Similarly, a significant expansion of proliferative CD4^+^ T cells was apparent in those who developed colitis but not in those who did not (Figure 3D). On the other hand, a subset of ICOS^+^ proliferative CD4^+^ T cells expanded significantly in both those who developed colitis and those who did not (Supplemental Figure 1F). Interestingly, we also found differences in Th1 cells. While the proportion of Th1 cells declined between baseline and on-treatment time points in the no-colitis group, there were no significant changes in those who developed colitis (Figure 3D).

We next wanted to compare the on-treatment immune profile of both colitis and no-colitis patients to determine whether an immunotherapy-induced imbalance could also be associated with colitis (Figure 3, A and B). This also showed differences in both innate and T cell populations (Figure 3, A and B, and Supplemental Figure 1); one of these differences was a significant reduction in Tregs in those who developed colitis (Figure 3D). In addition, those who developed colitis also had significantly higher levels of Th1 and proliferative CD4^+^ T cells (Figure 3D and Supplemental Figure 1F) on-treatment. Importantly, none of the major changes observed above were skewed by steroid treatment as 5 of 19 (26%) who received steroids in the colitis group did not show significant changes to these populations (Supplemental Figure 7, A and B). Together, these data demonstrate the multitude of systemic changes induced by combination immunotherapy; one of these changes was an expansion of proliferative T cells, including Th1 populations could be associated with colitis.

### Monocytic tissue infiltration is apparent in broader regions of the colon.

**Figure 4:**
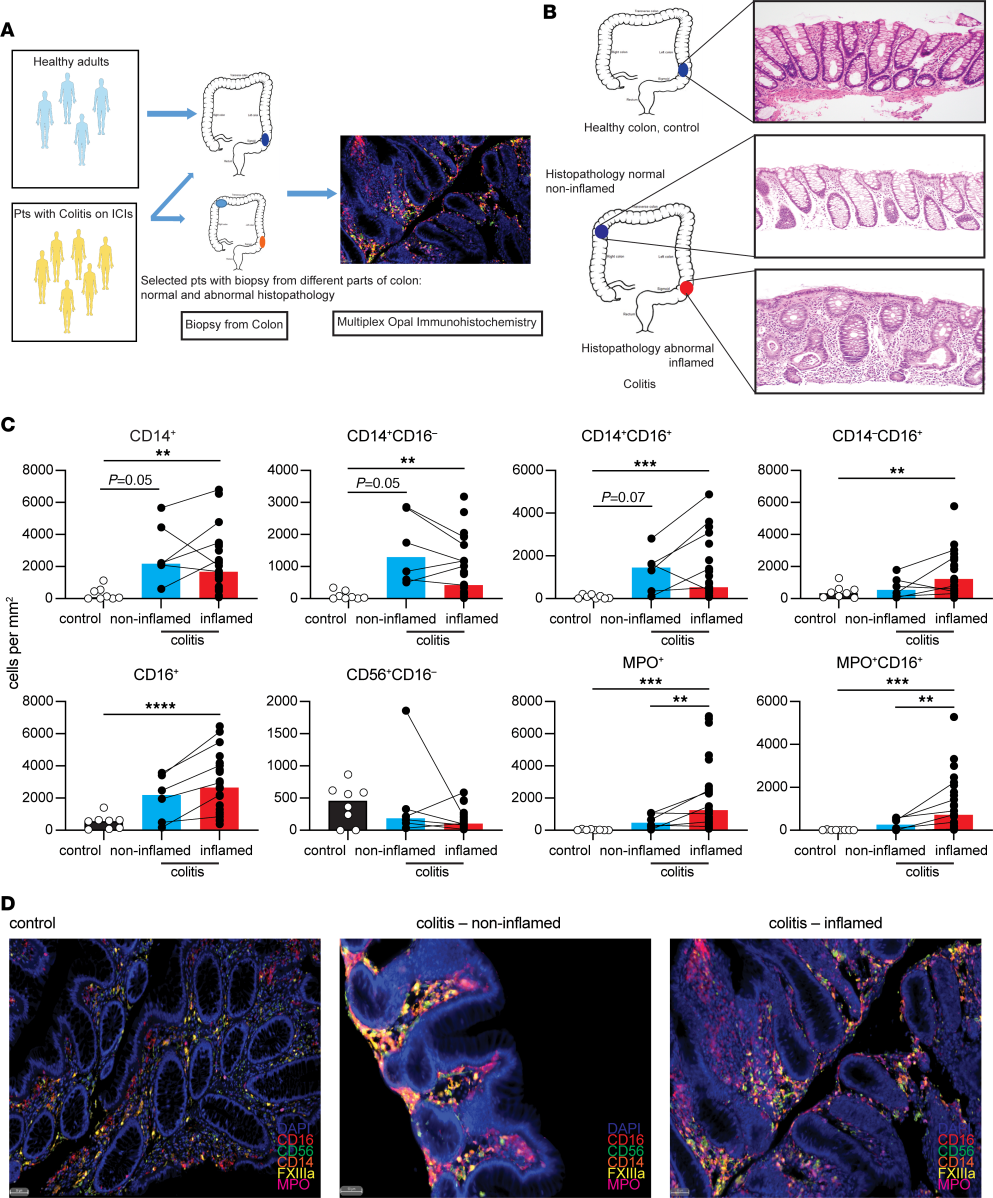
Myeloid cell subsets are altered at both histologically normal and diseased sites in colitis patients during toxicity. (**A**) Healthy donors (control) and patients with colitis had colon samples biopsied. A subset of colitis patients had paired biopsy from a endoscopically noninflamed colon (colitis_noninflamed) and endoscopically inflamed colon (colitis_inflamed). (**B**) Representative H&E staining at colon sites confirming differences between inflamed and noninflamed regions. (**C**) Myeloid cell subset counts in colon tissue. (**D**) Representative plots of mIHC staining from colon sections. Brown-Forsythe and Welch ANOVA with Dunnett T3 multiple-comparison test in 3 groups. Median shown. *P ≤ 0.05, **P ≤ 0.01, ***P ≤ 0.001, ****P ≤ 0.0001. Scale bars: 50 µm.

We next wanted to determine which immune populations within the colon were associated with immunopathology during colitis. In order to understand how immune cell recruitment could contribute to the development of colitis, we examined biopsy specimens taken from both endoscopically defined inflammatory regions (colitis_inflamed) and regions deemed endoscopically normal (colitis_noninflamed) from the same patient (Figure 4, A and C). Conventional histopathological analysis of the 2 regions (Figure 4B and Supplemental Figure 8) confirmed significant differences between inflamed and noninflamed biopsies, suggesting possible microscopic colitis in endoscopically normal regions. Normal healthy colon biopsies (control) were also used for comparisons. mIHC staining for myeloid cells showed increased infiltration of monocytes in both inflamed regions and noninflamed regions of the colon when compared with healthy controls (Figure 4D), suggesting that monocytic infiltration was likely occurring in the entire colon. We found accumulation of total CD14^+^ cells, CD14^+^CD16^+^ cells, and CD14^+^CD16^–^ cells even in noninflamed regions of the colon when compared with healthy controls, with levels comparable with those of the inflamed regions (Figure 4D). A similar increase in CD14^+^ monocytes was observed in the single-cell transcriptomic dataset by Luoma et al. (17) in the colon of no-colitis patients who received combination immunotherapy (Supplemental Figure 3A), although the differences were not statistically significant.

mIHC also revealed a significant increase in total CD16^+^ cells, as well as CD16^+^ macrophages, in both inflamed and noninflamed regions of colon in patients with colitis (Figure 4D), suggesting that infiltrating monocytes could have differentiated into CD16^+^ macrophages. The increase in CD16^+^ cells was highest within inflamed regions, including MPO^+^ neutrophils, suggesting that multiple innate populations were involved in the pathogenesis. A similar increase in CD16^+^ cells in colitis biopsies was found in the data of Luoma et al. (Supplemental Figure 3A) (17). Despite some differences in systemic NK cell populations, there were no significant increases in NK cells within noninflamed or inflamed colon (Supplemental Figure 3B). These data clearly demonstrate a monocytic infiltration of the whole colon, even outside of inflamed regions. The presence of increased CD16^+^ cells is also suggestive of a potential role for Fc effector functions.

### T cells specifically expand within clinically apparent inflamed lesions of the colon.

**Figure 5:**
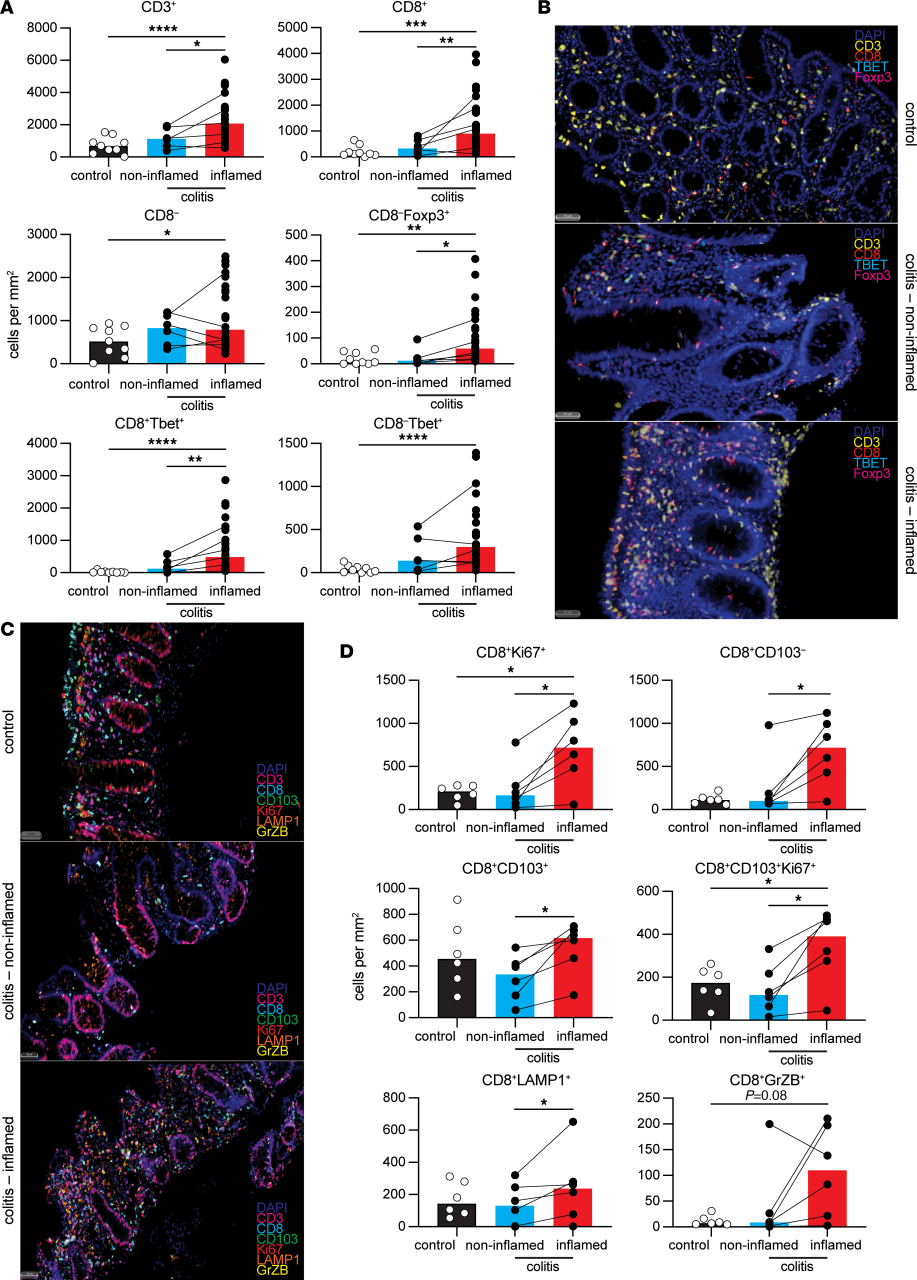
T cell subsets are selectively altered at diseased sites in patients with colitis during toxicity. (**A**) T cell subset counts in colon tissue. Brown-Forsythe and Welch ANOVA test in 3 groups were used. Median shown. *P ≤ 0.05, **P ≤ 0.01, ***P < 0.001, ****P < 0.0001. (**B**) Representative T cell staining across groups. (**C**) Representative CD8^+^ T cell stained with cytotoxicity markers across groups. (**D**) CD8^+^ T cell subset counts in colon tissue. Brown-Forsythe and Welch ANOVA with Dunnett T3 multiple-comparison test in 3 groups were used. Wilcoxon signed-rank test was performed in paired samples. Median shown. *P ≤ 0.05, control, healthy colon; colitis_noninflamed, histopathologically noninflamed colon from colitis patient; colitis_inflamed, inflamed colon from colitis patient. Scale bars: 50 µm.

Given the proposed mechanism of action of anti–PD-1 and anti–CTLA-4 inhibitors, we next wanted to determine the role of T cells within the colon. mIHC showed a significant accumulation of T cells within the regions of the colon showing clinically apparent colitis but not within the clinically noninflamed regions of the same patient (Figure 5), suggesting that T cell expansion was specific to inflamed tissue regions. This increase in T cells was largely due to CD8^+^ T cells; however, CD4^+^ T cells were also significantly increased compared with healthy controls (Figure 5A). Expanded CD8^+^ T cells expressed T-bet, granzyme B, and LAMP-1 and were proliferative (Figure 5, A–D), suggesting that they were effector cytotoxic T cells. In order to determine whether the expansion of CD8^+^ T cells was due to active recruitment or local expansion, we examined the tissue for the tissue-resident marker CD103. This showed that both CD103^+^ CD8^+^ and CD103^–^CD8^+^ populations were significantly increased within the inflamed regions of the colon when compared with noninflamed regions (Figure 5D and Supplemental Figure 3B) or healthy controls. Importantly, the majority of CD103^+^CD8^+^ Trm T cells also expressed the proliferative marker Ki67 (Figure 5D), suggesting that local activation within the inflamed regions could be driving the expansion of Trm T cells. Interestingly, Treg numbers (defined as CD3^+^CD8^–^FoxP3^+^) were also significantly increased within inflamed regions when compared with noninflamed regions or healthy controls (Figure 5A). Importantly, parallel to the systemic increase we saw in blood of proliferative CD4^+^ T cells, there was also a significant increase in Th1 cells (defined as CD3^+^CD8^–^T-bet^+^) within the inflamed regions of the colon when compared with noninflamed regions or healthy controls (Figure 5A). In order to validate the specific expansion of CD8^+^T-bet^+^ T cells within inflamed colon, we analyzed the single-cell transcriptomic data published by Luoma et al. (17) and also found a significant increase in the CD8^+^T-bet^+^ T cell subset in diseased colon (Supplemental Figure 3A). Together, these data demonstrate a critical role for type 1 immunity in the immunopathology of moderate-severe immunotherapy–induced colitis. The data also suggest a potential role for Trm T cells in propagating inflammation within the colon.

### Combination immunotherapy–associated changes linked to colitis are independent of tumor responses.

**Figure 6:**
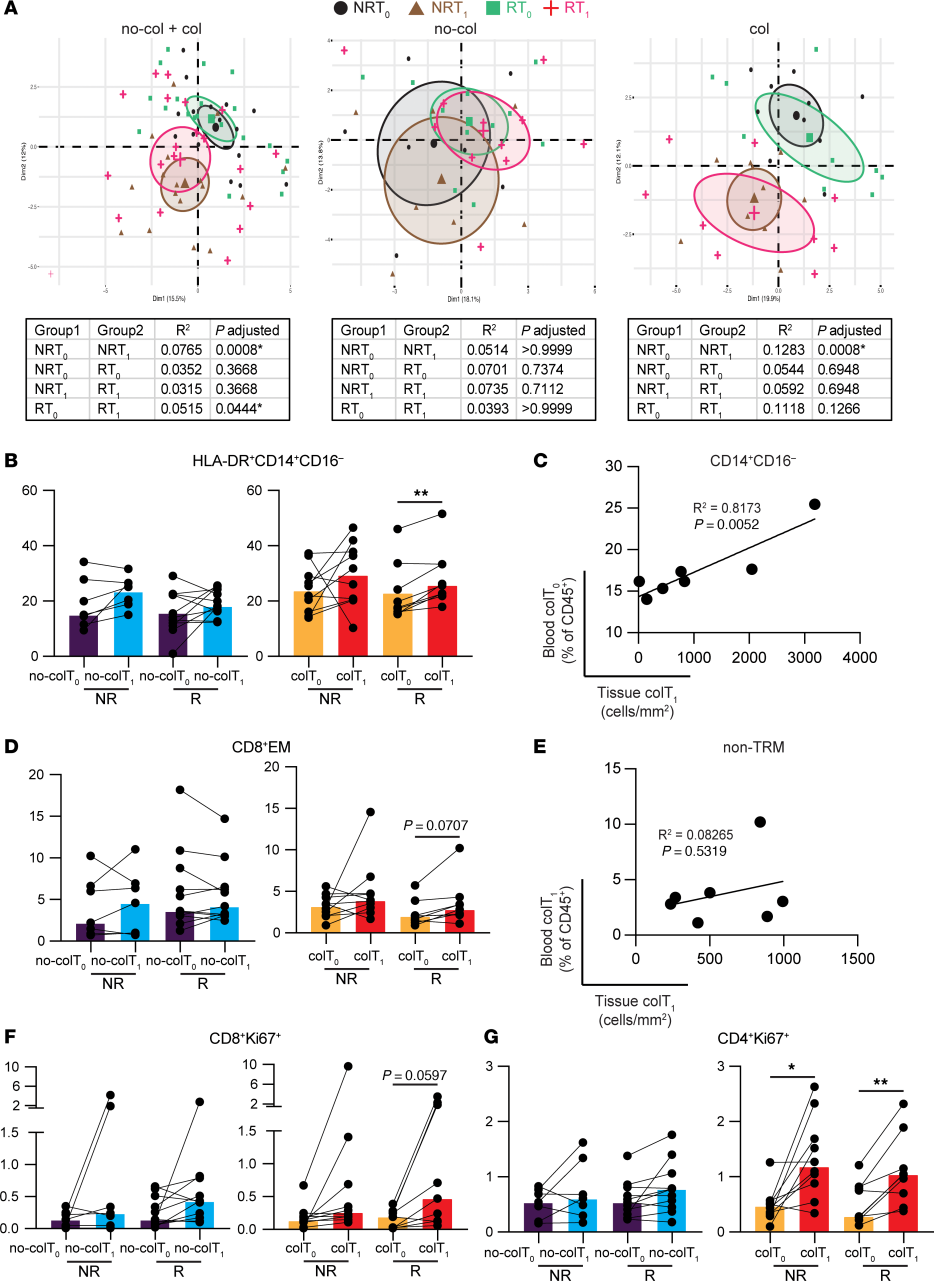
Colitis patients display unique immune cell repertoire independent of response status. (**A**) PCA plot based on clustering results of total PBMC across cohort. Plots were generated using all patients or split into no-colitis (no-col) or colitis (col) patients. Colors and shapes represent nonresponder baseline (NRT_0_), nonresponder treatment (NRT_1_), responder baseline (RT_0_), and responder treatment (RT_1_). PERMANOVA with multiple comparisons are shown in respective tables. (**B**, **D**, **F**, and **G**) Subset levels in noncolitis baseline (no-colT_0_), noncolitis treatment (no-colT_1_), colitis baseline (colT_0_), and colitis treatment (colT_1_) patients as NR or R. Brown-Forsythe and Welch ANOVA with Dunnett T3 multiple-comparison test (unpaired groups) or mixed-effects analysis with Šídák’s multiple-comparison test (paired time points) were used. Median shown. *P ≤ 0.05, **P ≤ 0.01. (**C** and **E**) Correlation plots with Pearson correlation, showing *P* and *R*^2^ values. Dots represent individual colitis patients with paired blood and tissue samples. (**C**) Comparison of blood at baseline and tissue at treatment. (**E**) Comparison of blood and tissue at treatment. EM, effector memory; TRM, tissue-resident memory.

We next wanted to determine which of the systemic and local changes associated with combination therapy is more likely to specifically contribute to the development of colitis. In order to determine whether response to immunotherapy or colitis was associated with the greatest immunotherapy-induced changes to the immune profile, we examined our cohort, which was composed of almost equal proportions of responders (20 of 37, 54%) and nonresponders (17 of 37, 46%). PCA and PERMANOVA of the full immune profile revealed that the most significant changes occurred in those who developed colitis on-treatment and that the response status did not fully differentiate the groups (Figure 6A). We then examined 56 immune populations individually at baseline and on-treatment between responders and nonresponders who did or did not develop colitis (4 groups: baseline responder, RT_0_; treatment responder, RT_1_; baseline nonresponder, NRT_0_; treatment nonresponder, NRT_1_). This also revealed that some of the changes were associated with colitis, irrespective of the response status (Supplemental Figures 5 and 6).

Focussing on the innate immune profile, the expansion of HLA-DR^+^CD14^+^CD16^–^ cells was observed in those who developed colitis, with values reaching significance in responders but not in nonresponders (Figure 6B). Importantly, there was also a significant correlation between baseline numbers of monocytes in blood and their numbers in the colon during colitis of paired samples (Figure 6C), suggesting that baseline monocytes are indeed likely to play a role in the development of colitis. These data reveal a potential role for the innate immune profile in determining susceptible individuals who are likely to develop colitis on combination therapy, irrespective of their response status.

We next examined the role of systemic T cells during response. There was no significant difference in the expansion of proliferative CD8^+^ T cells in the blood among those who developed colitis and responded to treatment compared with those who did not (Figure 6F), suggesting that it was not specific for the response status. Similarly, there was no difference in the TEM subset of CD8^+^ T cells between responders and nonresponders who developed severe colitis (Figure 6D). Importantly, when we determined the relationship between systemic T effector memory (TEM) and nonresident CD8^+^ T cells (defined as CD103^–^CD8^+^ T cells; non-Trm) within the colon, there was no correlation (Figure 6E), supporting the fact that expansion of CD8^+^ T cells within the colon could be largely driven by Trm T cells. Interestingly, the proliferative CD4^+^ T cell expansion was also seen in both responders and nonresponders who developed severe colitis, suggesting that this expansion was independent of the response status (Figure 6G). These data reveal that combination therapy–induced changes to the host immune profile are largely associated with colitis rather than an antitumor response.

## Discussion

Combination therapies are fast becoming the best option for enhancing the therapeutic efficacy of checkpoint blockade therapies. However, major barriers to their widespread use are irAEs. How combination therapies generally impact the host immune system and how they induce irAEs are not fully understood. Previous studies addressing this have focussed on either systemic responses or local responses (17, 19). In addition, studies thus far have not examined the immunological mechanisms that underlie response to immunotherapy against the development of irAEs. By analyzing both the systemic and tissue-specific changes on paired samples along with the response status, we show that the innate immune repertoire of individuals could play a key role in their susceptibility to developing adverse events. We also show that 3 potential pathways, including innate and adaptive immunity, could contribute to the development of one of the most troublesome irAEs: colitis. Fc effector function–mediated activation of monocytes and macrophages could precede the development of inflammatory foci within the colon, and these foci, in turn, are characterized by type I immunity and local expansion of Trm T cells. Importantly, we show that colitis-associated changes to the immune profile are not only independent of the response status, but are also greater in magnitude, highlighting the importance of irAEs when evaluating biomarkers for response.

Not everyone who is treated with checkpoint blockade therapy develops irAEs (20). Interestingly, prolonged treatment does not appear to increase the cumulative incidence of irAEs (21). Aside from vitiligo in patients with melanoma, the types of irAEs developing in patients undergoing immunotherapy are similar, regardless of the cancer type. These observations point to a possible group of susceptible individuals in whom the checkpoint blockade tips the balance toward autoreactivity, but what predisposes these individuals to this is unclear. Our data show that the innate immune profile prior to immunotherapy is indeed distinct in those who developed colitis on combination immunotherapy when compared with those who had no symptoms of colitis. We have identified classical monocytes as a potential innate population that is at a significantly higher proportion within the blood of those who go on to develop colitis. The differences in circulating monocyte numbers could be due to multiple factors, including gut dysbiosis, myelopoiesis, and influence of tumor itself (22, 23). Importantly, there was also monocytic infiltration of the colon outside of inflammatory lesions, with their numbers within the colon strongly correlating with monocyte numbers within the blood at baseline. Within the colon, monocytes are likely to differentiate to macrophages, and one of the key receptors expressed by these cells was CD16. This strongly suggests a potential role for Fc effector functions. IPI is an IgG1 antibody that can bind to Fc receptors efficiently. Such Fc-mediated activation could trigger a proinflammatory response from monocytes and macrophages, leading to secretion of IL-1, IL-6, and TNF-α. Indeed, single-cell RNA-Seq of colon tissues from patients who developed immunotherapy-induced colitis not only showed increased proinflammatory monocytes, but also showed increased expression of proinflammatory cytokines TNF-α and IL-1β (17). Therefore, from a biomarker development strategy point of view, it is likely to be an innate immune signature that could predict irAEs in treatments involving IPI. Interestingly, previous studies have implicated the same classical monocytes (HLA-DR^+^CD14^+^CD16^–^) as biomarkers of response to both anti–CTLA-4 therapy (24) and anti–PD-1 therapy (25). Both studies, however, did not consider irAEs, and it is likely the cohort included many patients who developed irAEs (such as colitis). This highlights the importance of incorporating irAE data when determining potential biomarkers of response. This could also explain why attempts to find biomarkers of response have been challenging and most findings thus far have failed to provide consistent results.

How combination immunotherapies enhance the incidence and severity of irAEs is not completely understood. Blocking CTLA-4 interactions are considered to induce a more robust costimulation, which in turn drives T cells with weak receptor affinity to respond, potentially paving the way for expansion of autoreactive T cells (26, 27). On the other hand, blocking PD-1 interactions is believed to reinvigorate dysfunctional immune populations (28). Another possible mechanism for irAE could be the disruption of immune regulation. Whether or not Tregs become depleted during immunotherapy remains to be elucidated. Early studies showed a clear reduction in Tregs in blood and melanoma tumors after IPI (anti–CTLA-4) treatment (24, 29). Recent studies examining the tumors and colon during immunotherapy-induced colitis, however, found increased Tregs at these sites (17, 30). The diversity of the gut microbiome has also been associated with irAEs, although how it relates to immune-mediated destruction of the end-organ remains to be seen (31).

Our data show that multiple changes affect the immune profile — including innate and adaptive immune populations — after combination immunotherapy, although the changes were more pronounced within the T cell compartment. Within the colon, there was a clear expansion of both CD4^+^ and CD8^+^ T cells, specific to the inflamed regions. The characteristics of these T cells suggested a strong bias toward type 1 immunity, with an expansion of T-bet^+^CD8^+^ T cells and T-bet^+^CD4^+^ T cells. Therefore, it is likely that immunotherapy-induced colitis is driven by type 1 immunity. IPI has been previously shown to induce a systemic expansion of CD4^+^ T cells (32), including Th1 effector cells (33). In our study, however, we found that systemic Th1 cells reduced on-treatment in those who did not develop colitis, while there was no significant change in those who developed colitis. It is possible that the systemic reduction was the result of tissue infiltration by Th1 cells, and in patients who developed colitis, it was balanced by further systemic expansion. In line with this, we found a greater expansion of proliferating CD4^+^ T cells in patients who developed colitis. Importantly, increased Th1 cells within the colon is consistent with recent findings where CXCR3 and its ligands CXCL10/CXCL9 were shown to be enhanced within the colon during immunotherapy-induced colitis (17). While the expansion of Th1 cells within the colon was likely the result of recruitment of cells from the blood, the expansion of CD8^+^ T cells was likely to be local. This is based on the following: (a) CD103^+^CD8^+^ T cells, which share the phenotype of Trm T cells, were proliferating within the inflammatory lesions, while expressing effector molecules and present in significantly increased numbers; (b) although there was an expansion of non-Trm T cells, there was no correlation between their number within the colon and the TEM CD8^+^ T cells in the blood of paired samples; and (c) a recent study that examined the TCR clonality showed that the majority of CD103^–^CD8^+^ T cells shared the same TCR clonotype as CD103^+^CD8^+^ T cells, suggesting that they likely originated from Trm T cells (17). Therefore, local expansion of Trm T cells is likely to constitute the vast majority of the expanded CD8^+^ T cells. Trm T cells express high levels of PD-1, and it is, therefore, likely that anti–PD-1 antibodies were responsible for their expansion within the colon. We and others have shown that anti–PD-1 therapy does expand CD103^+^CD8^+^ T cells (34).

The specificity of the T cell populations involved in the pathogenesis of colitis is likely to be against autoantigens or specific microbial antigens. It is possible that some of the Trm-like T cells within the colon are autoreactive but are kept quiescent by checkpoint receptors. Systemic treatment with anti–PD-1 could lead to the activation of these T cells, resulting in a breakdown of peripheral tolerance. A systemic expansion and recruitment of Th1 cells to the colon could also contribute to autoreactive T cells. Treatment with IPI has been shown to increase the diversity of the CD4^+^ T cell repertoire (13), leading to potential autoreactive populations that, in turn, contribute to the pathogenesis of colitis. While activation/reactivation of these autoreactive T cells could be a critical step in the development of irAEs, our data suggest that nonspecific activation of innate cell populations could exacerbate this process. Defining these pathways provides opportunities to develop specific therapeutic interventions. A recent study using preclinical models has shown that anti–CTLA-4 therapy activates microbiome-specific T cells in the skin (35). Therefore, it is also possible that the Trm population expanding in our patients is likely specific for gut microbial antigens.

The association between irAEs and response to immunotherapy is an interesting research focus. There is some evidence showing that gastrointestinal irAE is associated with favorable outcomes (36–39); however, our data show that both are unrelated. It is clear that at least some of the immunological changes that are induced by checkpoint blockade are specific for patients who develop colitis, irrespective of their response status. This shows potential opportunities to specifically target pathways associated with irAEs without compromising the response to immunotherapy. The current treatment options for irAEs are largely corticosteroid-based therapies as a first-line treatment and infliximab, vedolizumab, mycophenolate mofetil, and cyclosporin as second and/or third-line options. Understanding the immunological mechanisms that drive irAEs could explain why some treatments work in some but not all patients. It is likely that vedolizumab works by blocking the recruitment of systemically expanded Th1 cells by blocking the α4β7 integrin. There is emerging clinical evidence that supports the effectiveness of vedolizumab against immunotherapy-induced colitis (40). In addition, therapies targeting proinflammatory cytokines such as TNF-α and IL-6 (41) are likely to target innate responses.

There are some limitations to this study. Patients who did not develop colitis had slightly lower doses of IPI in combination with anti–PD-1. Sample collection time varied between patients in the colitis group, although the kinetics of treatment-related expansion or changes in T cells or myeloid cells were comparable. A comparison of colon tissue biopsies taken from patients treated with combination therapy without developing colitis would have further strengthened our data; however, such samples were not possible to obtain in this study (and would have required prospective ethical approval and informed patient consent). Our analysis on the possible link between colitis and the response status is also limited by the small cohort size.

This study demonstrates the immunological mechanisms that underlie the development of combination immunotherapy–induced colitis and how they relate to tumor response status. Our data suggest that the innate immune makeup of patients prior to the commencement of immunotherapy could play a role in their susceptibility to irAEs and may become a useful biomarker. Importantly, the study also demonstrates that immunotherapy-induced changes to the systemic immune repertoire that are associated with the development of colitis are present irrespective of tumor responses, suggesting that response and irAEs are likely independent and irAEs such as colitis can be treated without compromising the tumor control mechanisms.

## Methods

### Study design and samples.

Immune cell populations in peripheral blood and colon biopsy samples were studied from patients with melanoma treated with combination immunotherapy (anti–CTLA-4 antibody, IPI, combined with anti–PD-1 antibody, nivolumab or pembrolizumab, given concomitantly or sequentially) at Melanoma Institute Australia from April 2015 to April 2019.

For analyzing peripheral blood, we retrospectively identified a cohort of combination immunotherapy–treated patients based on similar baseline characteristics, outcomes, and sample availability. Nineteen of these patients developed immune-mediated moderate-severe colitis (grade ≥ 2–4, according to the Common Terminology Criteria for Adverse Events, CTCAE v.5.0) and required systemic steroid treatment on combination immunotherapy (42). They were defined as the colitis group. As a control group, 18 patients were retrospectively selected who received combination immunotherapy for melanoma and did not develop immune-mediated colitis/diarrhea of any grade or any other moderate-severe toxicities (≥grade 3) while on-treatment (≤grade 2 skin toxicity and endocrinopathy was allowed). They were defined as the no-colitis group. The PBMCs for both cohorts were collected for 2 time points: baseline before commencement of immunotherapy and then on-treatment. The median time to collect on-treatment PBMC samples for colitis group was 5.1 weeks (range, 1.5–15.3 weeks). The median time for collection of on-treatment PBMC samples for no-colitis group was 6.7 weeks (range, 3–14.1 weeks) to match the time points of the colitis group. The majority (14 of 19, 74%) of on-treatment PBMC (colT_1_) samples for colitis group (16 of 19, 84%) were collected before AED (median 15 days; range, 2–27 days). Five patients (5 of 19, 26%) had PBMC collected after AED (median 8 days; range, 1–27 days). These patients were on systemic steroid treatment when the PBMCs were collected. The baseline characteristics of the colitis and no-colitis patients are summarized in Supplemental Table 1. The median age was 64 years (range, 47–74 years) in the colitis group and 58 years (range, 34–79 years) in the no-colitis group with male predominance in both groups. Most of the patients in both groups had stage IV melanoma (95% in colitis group, 83% in no-colitis group). Patients of both cohorts received immunotherapy anti–CTLA-4 antibody (IPI) and anti–PD-1 antibody (nivolumab or pembrolizumab, PD-1) as combination or sequentially. In the colitis group, 13 (68%) patients received standard doses of IPI (3 mg/kg) + PD-1 (1 mg/kg) as combination (IPI [3 mg/kg] + PD-1 [1 mg/kg] every 3 weeks for 4 doses followed by PD-1 [3 mg/kg or flat dose 240 mg] every 2 weeks). Two patients (11%) received standard sequential treatment (IPI [3 mg/kg] every 3 weeks for 4 doses followed by PD-1 [3 mg/kg] every 2 weeks). Two patients (11%) had combination treatment IPI (1 mg/kg) +PD-1 (2 mg/kg) (IPI [1 mg/kg] + PD-1 [200 mg] every 3 weeks for 4 doses followed by PD-1 [200 mg] every 3 weeks), 1 patient (5%) had low dose IPI + PD-1 (IPI [50 mg every 6 weeks or 100 mg every 12 weeks] + PD-1 [200 mg] every 3 weeks), and 1 patient (5%) had IPI (3 mg/kg) + PD-1 (3 mg/kg) (IPI [3 mg/kg] + PD-1 [3 mg/kg] every 3 weekly for 4 doses followed by PD-1 [3 mg/kg] every 2 weeks) under clinical trial protocols (43–45). Out of 18 patients in the no-colitis group, 4 patients (22%) had IPI (3 mg/kg) + PD-1 (1 mg/kg). Nine patients (50%) had IPI (1 mg/kg) + PD-1 (2 mg/kg) regimen, and 5 patients (28%) had low dose IPI + PD-1 under the clinical trial protocol. The majority (15 of 19, 79%) of the patients in the colitis cohort had grade 3–4 colitis. For patients in the colitis group, the date of first reported/documented symptom of colitis was defined as adverse event date (AED). The median time to develop colitis in the colitis group was 5.1 weeks (range, 1.5–15.3 weeks). The objective response rate (ORR) for each cohort was determined as per RECIST v1.1. Patients with a complete response (CR), partial response (PR), or stable disease (SD) of greater than 6 months were defined as responders and comprised 47% (9 of 19) of colitis group and 61% (11 of 18) of no-colitis group. Patients who had progressive disease (PD) or SD for less than or equal to 6 months were defined as nonresponders; this group was composed of 53% (10 of 19) of colitis and 39% (7 of 18) of no-colitis cohorts. Other significant immune-mediated toxicities in the colitis group were rash (21%), vitiligo (16%), hypophysitis (16%), thyroid disorders (21%), pneumonitis (16%), and hepatitis (21%). One patient (5%) had immune-mediated diabetes mellitus. For the no-colitis cohort, 3 patients (17%) had rash, 2 (11%) had vitiligo, and 2 (11%) had thyroid disorders (Supplemental Table 1). PBMC samples were comprehensively analyzed in both groups by mass cytometry using a customized panel of 41 metal isotope–conjugated antibody markers to identify major immune cell populations and their subtypes (Supplemental Tables 2 and 3).

For analysis of peripheral tissue, colon biopsy (formalin-fixed paraffin-embedded [FFPE]) tissue from 26 patients who developed histologically confirmed moderate-severe colitis on IPI + PD-1 were examined with mIHC. Twelve patients of 26 in this cohort were also included in the PBMC colitis group. During colonoscopy, biopsies were taken from multiple sites of colon. A subset of colitis patients (*n* = 7) had biopsies from endoscopically and histopathologically abnormal site (colitis_inflamed) and a biopsy from endoscopically and a histopathologically normal site (colitis_noninflamed). We analyzed these paired biopsy samples with mIHC (Figure 3, A and B). A cohort of healthy adults (*n* = 9) undergoing a screening colonoscopy were collected as healthy controls. The characteristics of patients are summarized in Figure 3C. The median age of healthy controls was 36 years (range, 27–67 years) and colitis cohort (*n* = 26) was 62 years (range, 30–74 years). The majority of the patients in colitis cohort were male (17 of 26, 65%). The colitis patients received immunotherapy either as standard combination of IPI (3 mg/kg) + PD-1 (1 mg/kg) (22 of 26, 85%) or sequentially IPI followed by PD-1 therapy (35%). Most of the patients developed grade 3 colitis (23 of 26, 88%) following a median of 6.5 weeks (range, 1.5–18.4 weeks) from the first cycle of immunotherapy. The median time to perform colonoscopy was 8 days from onset of symptoms (range, 0–37 days), and patients were on steroids while the biopsies were taken. In the colitis cohort, recto-sigmoid colon was the most common site of biopsy (9 of 19, 47%), followed by descending (7 of 19, 37%) and right colon (including terminal ileum, 2 of 19, 16%). Of the 7 patients who had paired biopsies, most of the abnormal biopsies were from descending colon (4 of 7, 57%) followed by recto-sigmoid (2 of 7, 29%) and right colon (1 of 7, 14%). The histologically normal biopsies in these patients were taken from recto-sigmoid (3 of 7, 43%) and right colon, including terminal ileum (3 of 7, 43%) and descending colon (1 of 7, 14%).

### Mass cytometry sample preparation.

The blood samples were processed to isolate PBMCs by gradient density centrifugation, using Ficol density gradient centrifugation (600*g*, room temperature, 30 minutes) of whole blood (Stem Cell Technologies). Single-cell suspensions were then cryopreserved in FBS supplemented with 10% DMSO (Sigma-Aldrich), using a controlled freezing unit (Cool Cell LX) and stored in liquid nitrogen for later use.

The cryopreserved PBMC vials were resuscitated for mass cytometry analyses by rapid thawing and slow dilution in warm RPMI-1640 (Thermo Fisher Scientific) medium supplemented with 10% FBS and 1:10,000 universal nuclease (Thermo Fisher Scientific). Cells were then counted using Trypan blue exclusion viability dye, and they were washed in FACS buffer (1× DPBS supplemented 1% FBS and 0.05% EDTA), ready for downstream applications.

### Mass cytometry antibodies.

For Immunophenotyping of PBMCs, 41 metal-tagged and 5 fluorophore-tagged monoclonal antibodies were optimized and employed (Supplemental Tables 2 and 3). Antibody specificities were chosen to provide comprehensive coverage of T cells, myeloid cells, and NK cell phenotypic markers. Details of the metal and fluorophore-tagged antibody list are provided in Supplemental Table 2. All 41 metal-tagged antibodies were validated, pretitered, and supplied in pretest volume by the Ramaciotti Facility Reagent bank (University of Sydney, New South Wales, Australia). Reagent bank antibodies were either purchased from Fluidigm (Fluidigm) in preconjugated form, or unlabeled antibodies were purchased in a carrier-protein–free format and conjugated at the Ramaciotti Facility with the indicated metal isotope using MaxPAR conjugation kit (Fluidigm) according to the manufacturer’s protocol. For 5 markers, fluorophore-conjugated antibodies were used as primary antibodies, followed by secondary labeling of anti–fluorophore metal–conjugated antibodies (Supplemental Tables 2 and 3).

For staining, the samples were plated in 96-well U-bottom plates, followed by centrifugation at 500*g* for 5 minutes at room temperature. Following removal of supernatant, cell pellets were resuspended in 100 μL cisplatin (1:4,000 dilution in RPMI, Fluidigm) for 3 minutes at room temperature (20°C–24°C) to discriminate live from dead cells. The reaction was quenched by addition of 100 μL of RPMI. Barcoding was performed by incubating cells for 30 minutes with CD45 antibodies conjugated with various metals. After 2 washes with FACS buffer (PBS, 0.02% Sodium Azide, 0.5% BSA and 2 mM EDTA), differently CD45-labeled samples were then combined and resuspended in the first antibody cocktail containing fluorophore-conjugated surface antibodies and left to incubate 4°C for 30 minutes. Cells were washed 2 times in FACS buffer, resuspended in the second metal-conjugated surface antibody cocktail, and incubated for 30 minutes at 4°C. Following 2 washes with FACS buffer, cells were permeabilized and fixed using BD Pharmagen Transciption Factor Buffer Set (BD Biosciences) at 4°C for 40 minutes and stained with fluorophore-conjugated intracellular antibodies for 40 minutes. Cells were then washed in Perm/Wash buffer (1:20 dilution, eBiosciences) and suspended in the metal-conjugated intracellular antibody cocktail for 40 minutes at 4°C. Cells were then washed 2 times in Perm/Wash buffer and 1 time in FACS buffer, followed by fixation in 4% paraformaldehyde solution containing DNA intercalator (0.125 μM iridium-191/193; Fluidigm); they were incubated overnight at 4°C. After multiple washes with Milli-Q water (MilliporeSigma), cells were diluted to 800,000 cell/mL in Milli-Q water with 1:10 diluted EQ beads (Fluidigm) and filtered through a 35 μm nylon mesh. Cells were acquired at a rate of 200–400 cells/second using a CyTOF 2 Helios upgraded mass cytometer (Fluidigm).

Samples were stained and run in 6 batches. The samples from patients with 2 different time points were barcoded and run in the same batch. Each batch also included a batch control of a replicate aliquot of PBMC from a healthy donor. All .fcs files obtained from the Helios analysis were normalized using a processing function within the CyTOF Software (Fluidigm) based on the concurrently run EQ 4 element beads.

### Analysis of mass cytometry data.

Data analysis was performed using FlowJo version 10.6.1 software (FlowJo). Samples were pregated by identification of cells (to exclude beads), their staining of DNA intercalator, live/dead stain (cisplatin), and the expression of CD45 before exporting data for further analysis. Manual gating was performed, and major cell populations were identified as shown in Supplemental Figure 2. Gates were adjusted on the basis of batch-to-batch variations made apparent from the batch control data.

Computational analyses were done within R (46). FlowSOM clustering (47) was done using the R package Spectre (48). PCA was performed through Spectre, using scaled data with the prcomp function as part of the stats R package. To calculate differences between groups, a PERMANOVA was done using the package vegan (49). Permutational tests are powerful nonparametric tests, as they do not assume normal distribution or homogeneity of variance and only assume that data are exchangeable (50). Data were first scaled (to allow balanced comparisons between parameters) with the Euclidean distance calculated between points. In total, 4,999 permutations were done to generate *P* values, to provide power and confidence for α = 0.01 (51). For pairwise comparisons, the R package pairwiseAdonis was used with Holm’s correction for multiple comparisons (52).

For visualization, uniform manifold approximation and projection (UMAP) plots were generated in Spectre using the umap package (53). These were generated after FlowSOM clustering using downsampled data of 70,000 cells. For total cells, FlowSOM clustering and UMAP plots used CCR3, CCR6, CCR7, CD1c, CD3, CD4, CD8a, CD10, CD11b, CD11c, CD14, CD16, CD19, CD24, CD25, CD33, CD45RA, CD45RO, CD56, CD66b, CD68, CD117, CD141, CD161, CTLA-4, CXCR3, CXCR5, EOMES, Foxp3, GATA3, granzyme B, HLA-DR, ICOS, Ki67, PD-1, PD-L1, RORγt, T-bet, and TCRγδ. For myeloid cells, FlowSOM clustering and UMAP plots used CCR3, CCR7, CD11b, CD11c, CD14, CD16, CD45RA, CD45RO, CD56, CC66b, CD68, CD117, CTLA-4, granzyme B, HLA-DR, ICOS, Ki67, and T-bet. For T cells, FlowSOM clustering and UMAP plots used CCR7, CD3, CD4, CD8a, CD16, CD25, CD45RA, CD45RO, CD56, CXCR3, CXCR5, Foxp3, GATA3, granzyme B, Ki67, RORγt, T-bet, and TCRγδ. Manual gating was then used to confirm any changes identified by the unsupervised analyses (54).

### Opal mIHC staining.

Sections (4 μm) were produced from FFPE of colon biopsy samples and mounted on Superfrost Plus slides (Thermo Fisher Scientific). FFPE sections were heated in the oven at 65°C for 20 minutes, deparaffinized in xylene (5 minutes twice in xylene), and rehydrated in ethanol (5 minutes in 2 × 100% ethanol, 5 minutes in 95% ethanol, 5 minutes in 70% ethanol). Antigen retrieval was performed in AR9 buffer (Perkin Elmer, AR900) in the Decloaking Chamber (Biocare Medical) at 110°C for 10 minutes. Slides were cooled to room temperature in a water bath before staining. All staining was performed using an Autostainer plus (DAKO) or an intelliPATH FLX Automated Slide Stainer (Biocare Medical).

For the T cell panel, tissue sections were blocked with 3% H_2_O_2_ (Sigma-Aldrich) diluted in TBST for 5 minutes and then incubated with primary mouse antibody for CD3 (Cell Marque Corp, MRQ-39, 1:2,000), FOXP3 (Abcam, 1:2,000), T-bet (Cell Signaling Technology, 1:1,000), or CD8 (DAKO, 1:1,500) made up in Antibody Diluent/Block (Perkin Elmer) for 30 minutes. Slides were then incubated with either Opal Polymer HRP Ms + Rb (Perkin Elmer) for 10 minutes or Mach 3 Mouse Probe antibody or Mach 3 Rabbit Probe antibody (Biocare Medical) for 5 minutes and then Mach 3 Mouse HRP antibody or Mach 3 Rabbit HRP antibody (Biocare Medical) for 5 minutes (Supplemental Table 3 and Supplemental Figure 4). Afterward, slides were incubated with Opal fluorophore diluted in TSA (Perkin Elmer, 1:100); then, staining was repeated from antigen retrieval onward to remove the previous antibody and add subsequent antibodies in the panel. Single-color control slides were stained alongside the test panel to determine background staining and to create a library for later spectral unmixing. After staining with the final antibody, slides were incubated with Spectral DAPI (Perkin Elmer, 1:2,000) diluted in TBST for 5 minutes and coverslipped using Prolong Diamond Antifade Mountant (Thermo Fisher Scientific).

Samples were stained with the T cell cytotoxicity panel in the same manner. This included the sequential staining of FFPE tissue sections with the primary mouse antibody for Granzyme B (DAKO, 1:100), primary mouse antibody for CD103 (Abcam, 1:1,500), primary mouse antibody for CD8 (DAKO, 1:1,500), primary mouse antibody for LAMP-1 (Cell Signaling Technology, 1:1,000), primary mouse antibody for CD3 (Cell Marque Corp., 1:2,000), and primary mouse antibody for Ki67 (Cell Signaling Technology, 1:2,000). Slides were then counterstained with DAPI (1:2,000) and coverslipped as described previously.

The same protocol was repeated for the Myeloid panel. The FFPE tissue sections were sequentially stained with primary rabbit antibody for CD14 (Cell Marque Corp., 1:100), primary rabbit antibody for MPO (DAKO, 1:2,500), primary mouse antibody for FXIIIa (Cell Signaling Technology, 1:5,000), primary mouse antibody for CD56 (Cell Marque Corp., 1:500), primary mouse antibody for CD16a (Abcam, 1:800), and DAPI counterstain (Supplemental Tables 2 and 3).

### Multispectral imaging and analysis.

All mIHC imaging was performed using the Vectra 3.0.5 Automated Quantitative Pathology Imaging system (Perkin Elmer). Multispectral images (magnification, 20×) covering the entire colonic biopsy tissue were acquired using the DAPI, FITC, Cy3, Texas Red, and Cy5 fluorescent channels. Spectral unmixing of multispectral images was performed in inForm v2.4.2 (Akoya Biosciences) based on the signals acquired from single-color controls.

Multispectral images were exported for image analysis in HALO v2.3 (Akoya Biosciences) and stitched together to create a single high-resolution multispectral image for each tissue biopsy, upon which the analysis was performed. An algorithm was developed to detect individual cells based on nuclear DAPI staining. Positivity thresholds were set for each marker based on staining intensity. Cell phenotyping was performed based on cell marker expression as outlined in Supplemental Tables 4 and 5. Data for each cell phenotype, including cell count and marker expression, were exported from HALO.

### Statistics.

Statistical analysis and graphical representation were performed using GraphPad Prism, version 8.0 (GraphPad Software), unless specified elsewhere. Parametric and nonparametric tests were performed to compare groups. For comparisons between unpaired time points (including non-colT_0_ to colT_0_ and non-colT_1_ to colT_1_), a Brown-Forsythe and Welch 1-way ANOVA with Dunnett T3 multiple-comparison test was done. For comparisons between paired time points (including non-colT_0_ to non-colT_1_ and colT_0_ to colT_1_), a mixed-effects analysis with Šídák’s multiple-comparison test was done. For IHC comparisons between control, noninflamed colitis, and inflamed colitis, a Brown-Forsythe and Welch ANOVA with Dunnett T3 multiple-comparison test was done. A Pearson correlation was used for correlation plots.

### Study approval.

The study was conducted in accordance with Declaration of Helsinki, and written informed consent was obtained from all patients at the Melanoma Bio specimen Tissue Bank, Melanoma Institute Australia, with ethical approval from the Sydney Local Health District Human Research Ethics Committee (protocol no. X17-0312 & 2019/ETH07604).

## Author contributions

UP and AMM were involved in the design, intellectual oversight, and supervision of the study; KJN, TNG, ALF, and RA performed experiments; KJN, FMW, and CQ contributed to data analysis; RVR, IPDS, GM, JSW, RAS, and GVL contributed to supervision and integration of clinical data; KJN and FMW prepared the first draft of the manuscript; IPDS, GM, RAS, GVL, AMM, and UP provided intellectual input and helped prepare the manuscript; and ST, CJK, NS, and MSC provided patient samples. All authors also contributed to the revision of the manuscript. The order of the co–first and co–senior authors was determined based on the contribution to the workload.

## Supplementary Material

Supplemental data

## Figures and Tables

**Table 1:**
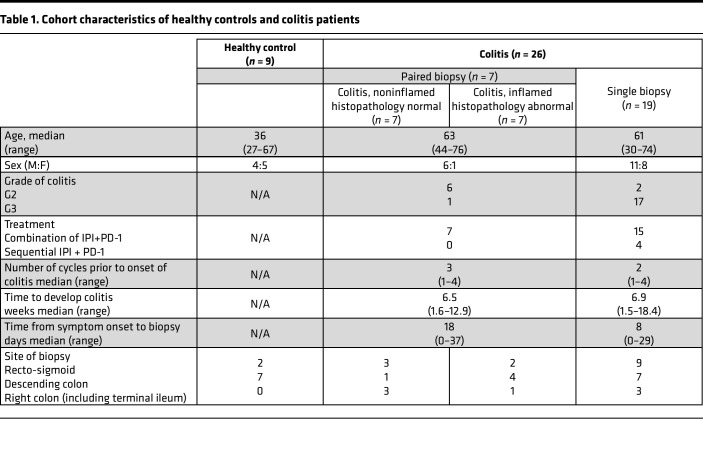
Cohort characteristics of healthy controls and colitis patients

## Version 1 09/29/2022

In-Press Preview

## Version 2 11/08/2022

Electronic publication
