# Supplementary material for: Distinct pretreatment innate immune landscape and posttreatment T cell responses underlie immunotherapy-induced colitis
Source: JCI Insight. 2022 Nov 8;7(21):e157839. doi: 10.1172/jci.insight.157839 (PMC9675442; doi:10.1172/jci.insight.157839)
Supplement: Supplemental data [file jciinsight-7-157839-s183.pdf]

Supplementary Figure 1

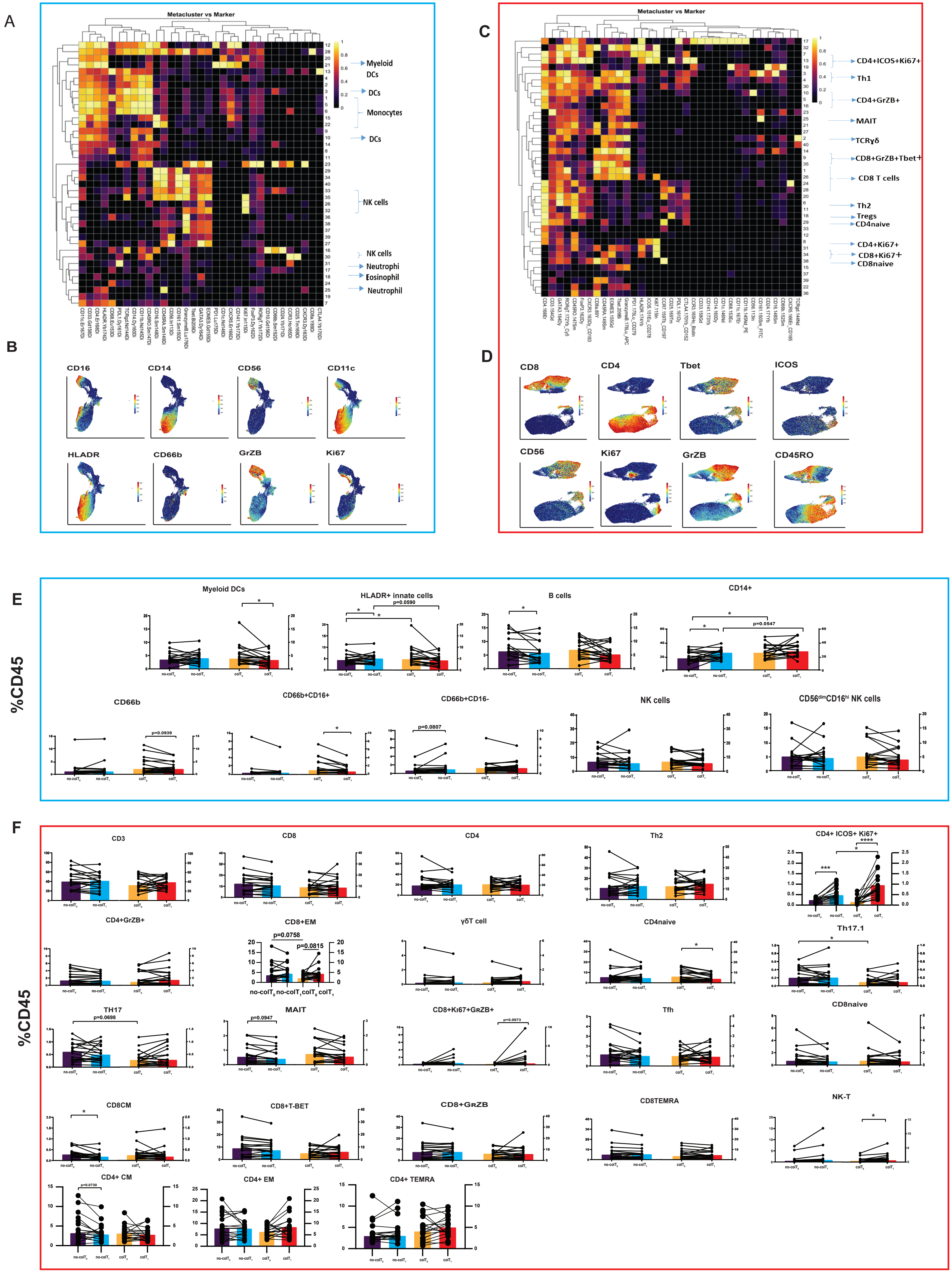

Supplementary Fig: 2

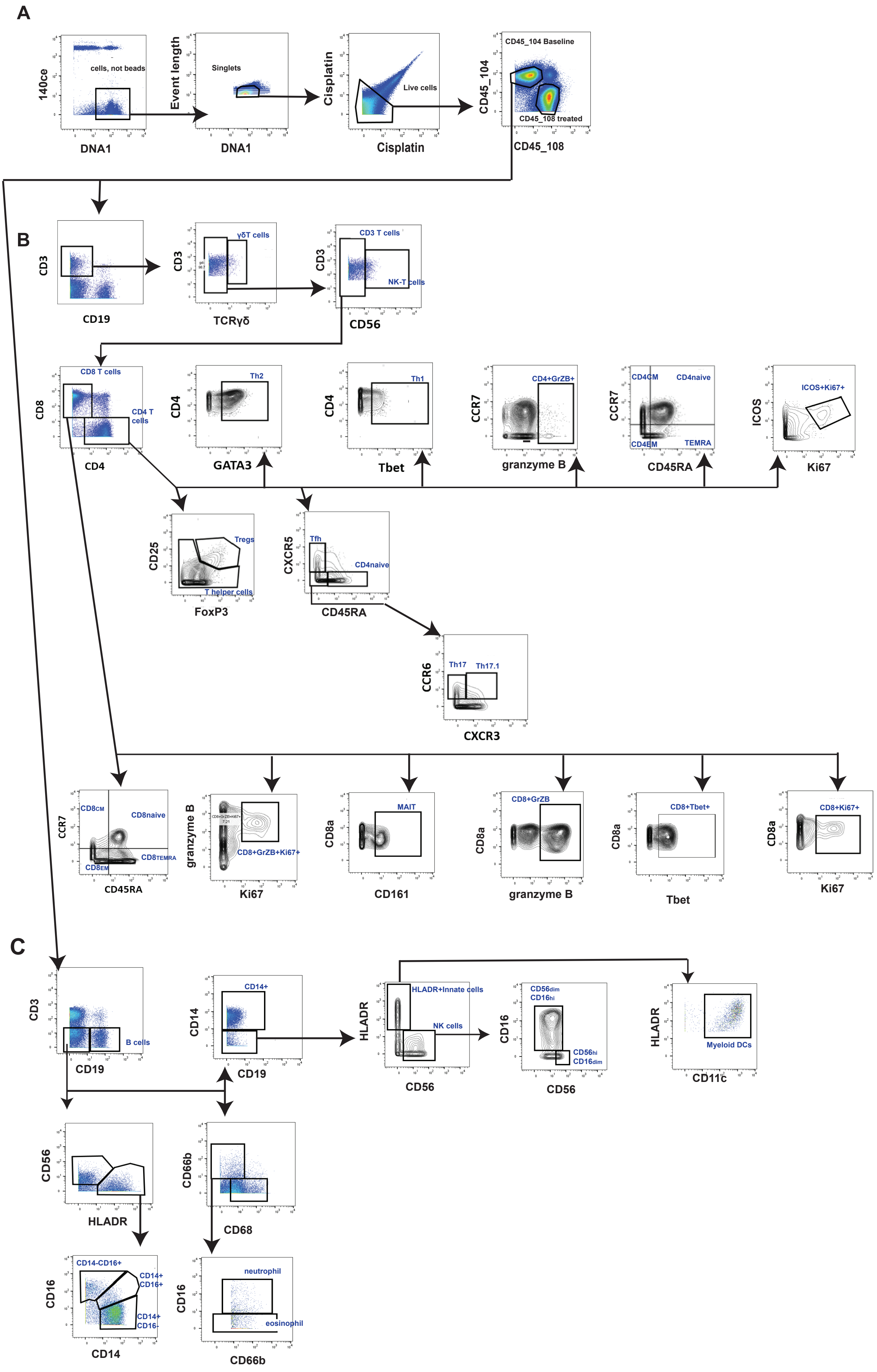

Supplementary Figure 3

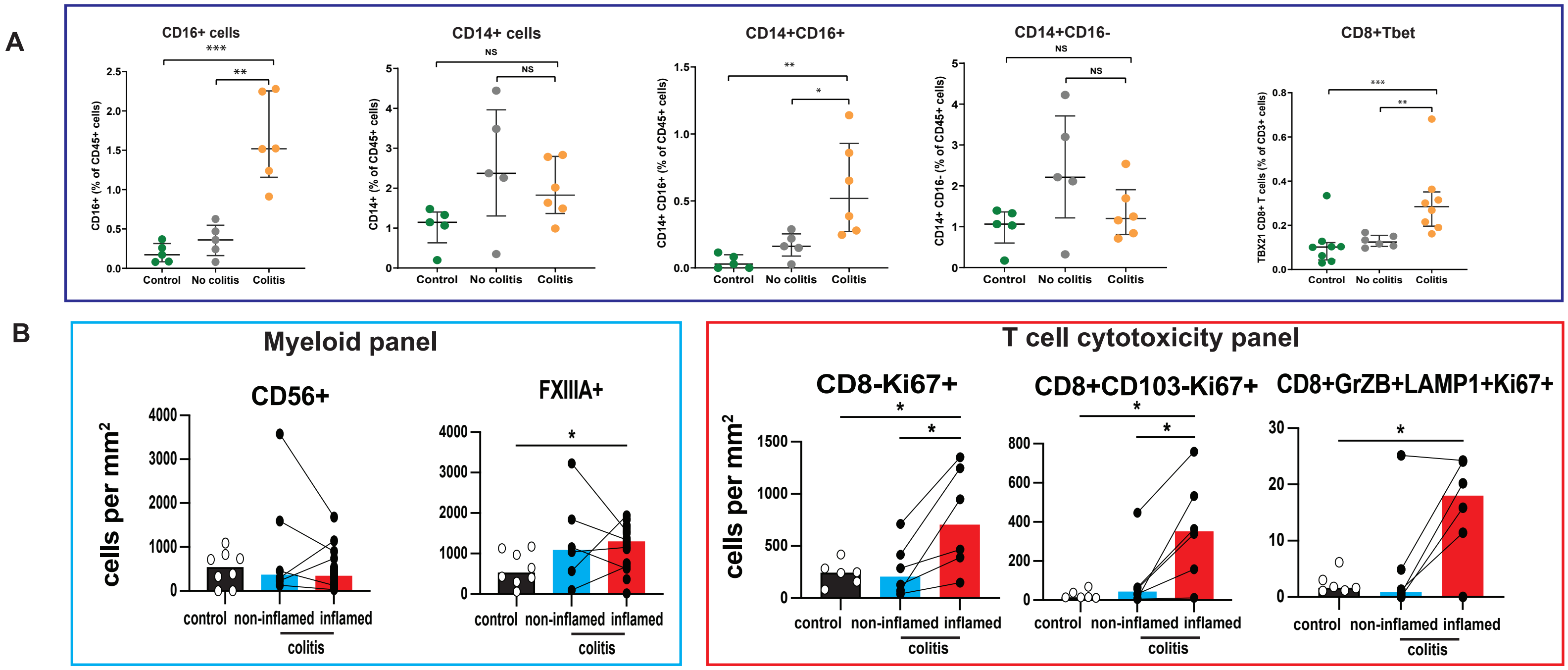

Supplementary Figure 4

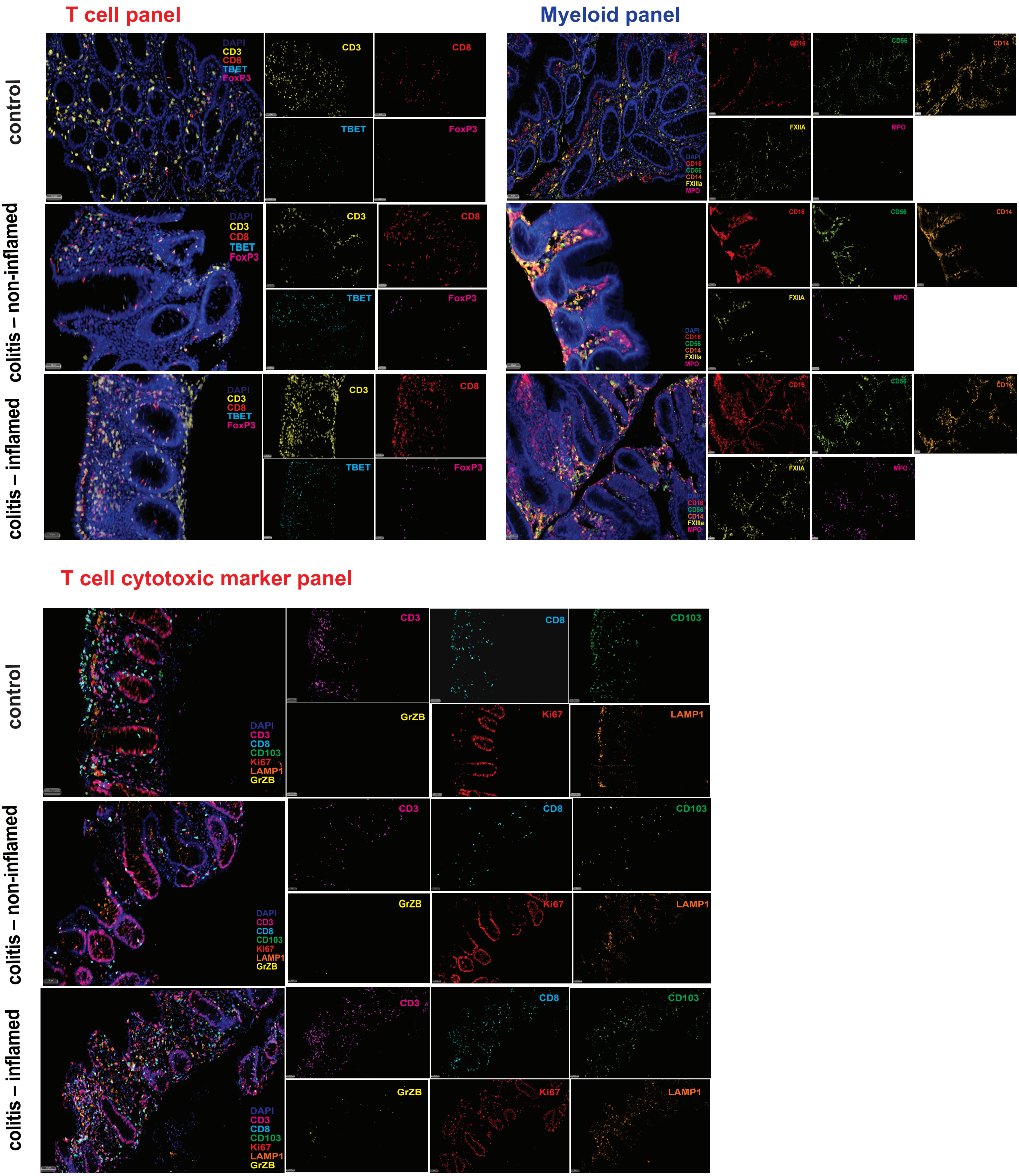

Supplementary Figure 5

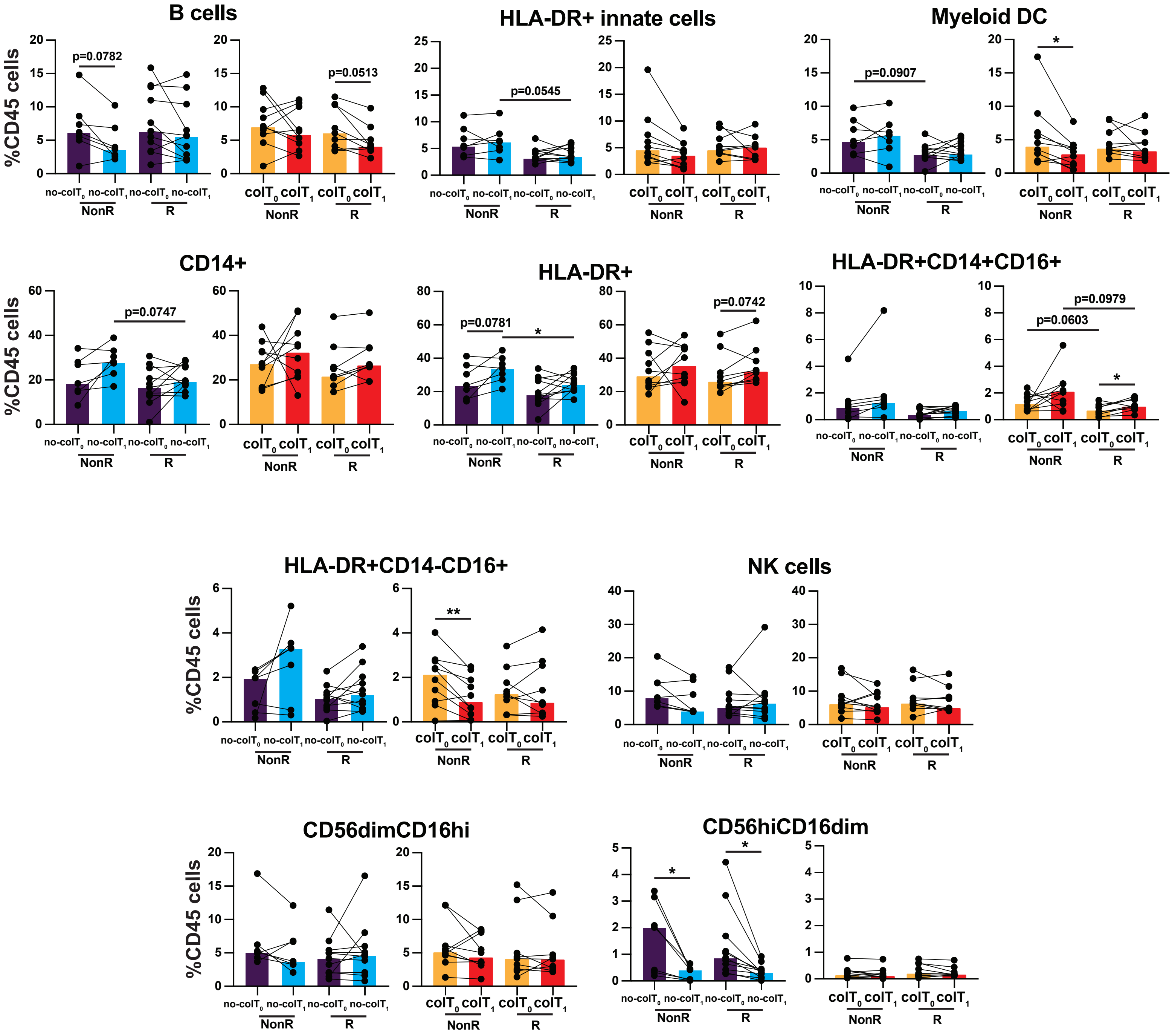

Supplementary Figure 6

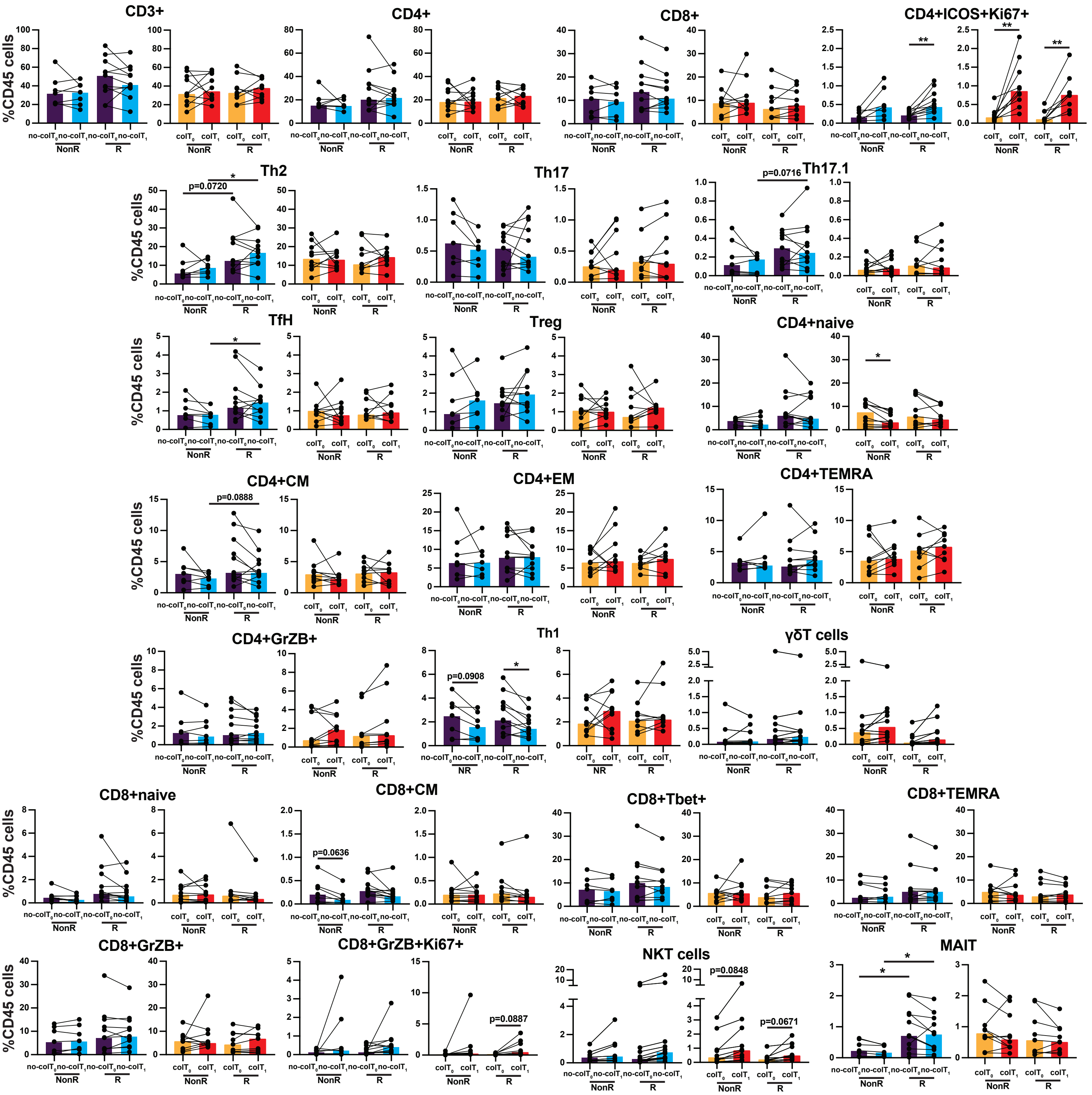

Supplementary Figure 7

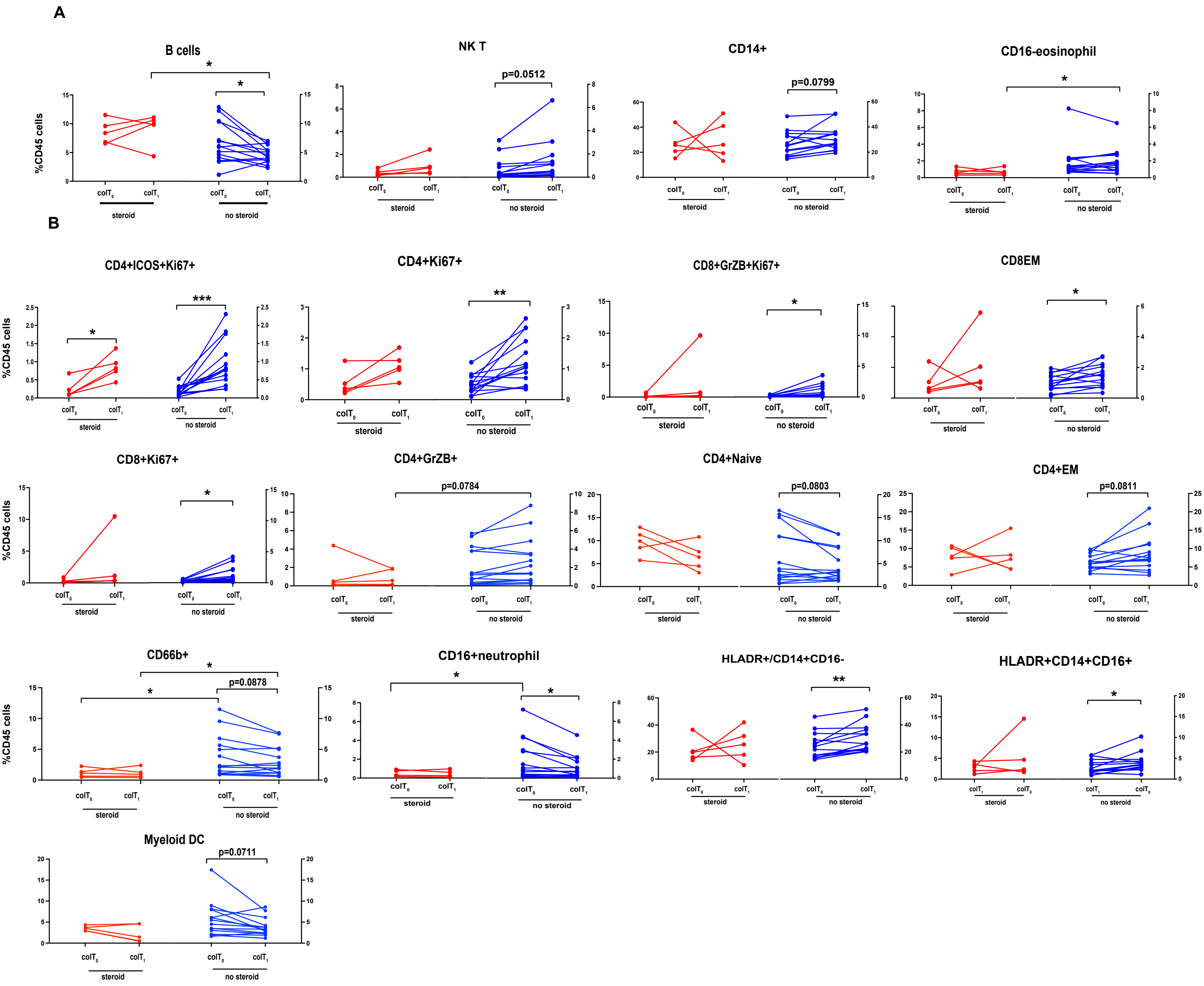

Supplementary Figure 8

A

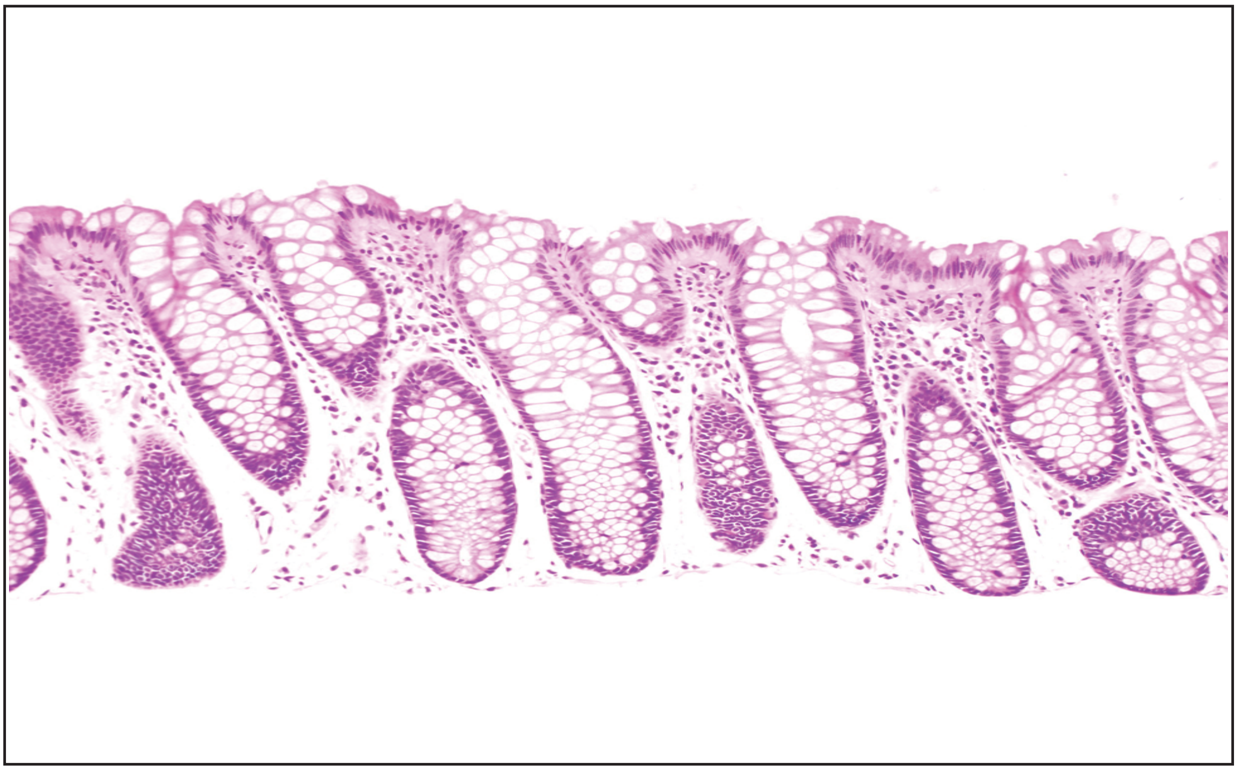

B

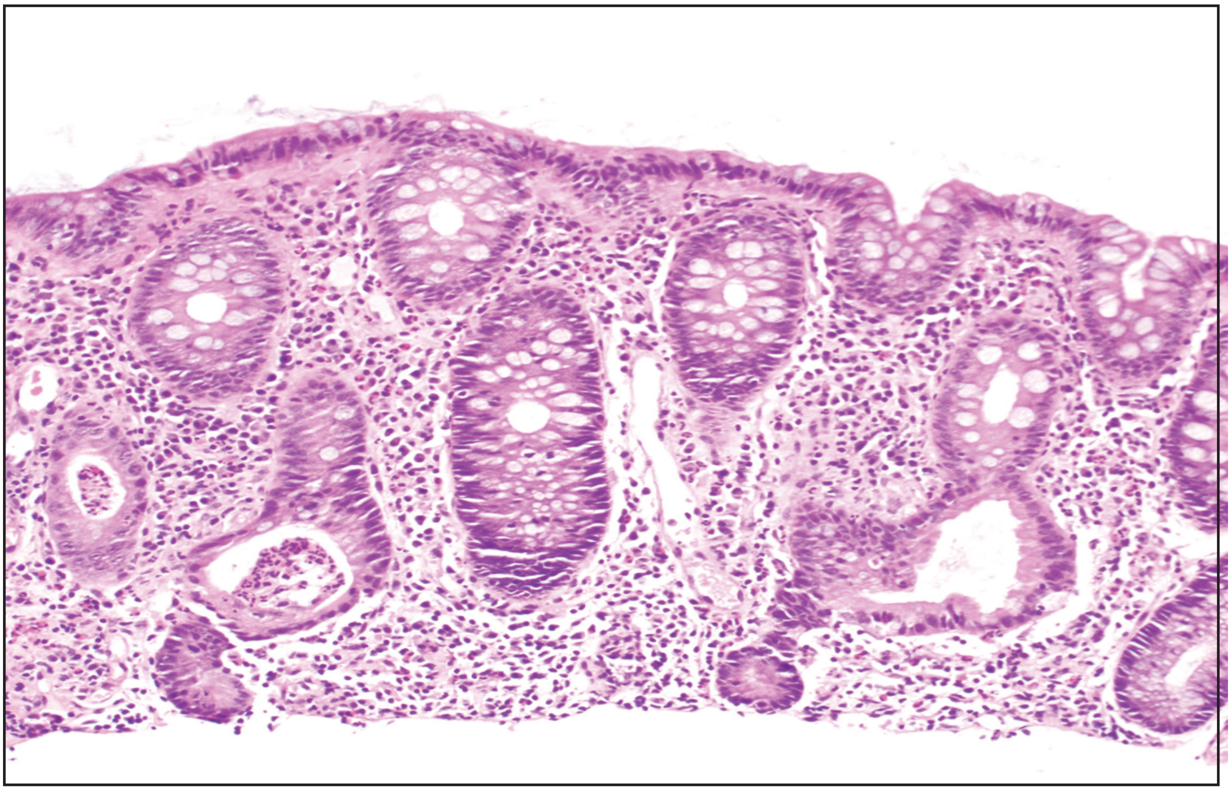

	colitis (col)<br>(n =19)	non-colitis (no-col)<br>(n =18)
Age, median<br>(range)	64<br>(47-74)	58<br>(34-79)
Sex (M:F)	13:6	12:6
AJCC v8 staging, n (%)		
Stage IIIC	1 (5)	3 (17)
Stage IV	18 (95)	15 (83)
Treatment, n (%)		
Combination CTLA-4 + PD-1	17 (89)	18 (100)
IPI(3)+PD1(3)	1 (5)	0 (0)
IPI(3)+PD1(1)	13 (68)	4 (22)
IPI(1)+PD1(2)	2 (11)	9 (50)
Low dose IPI+PD1	1 (5)	5 (28)
Sequential CTLA-4, PD-1	2 (11)	0 (0)
No of cycles received, median (range)	2<br>(1-4)	4<br>(2-4)
Time to develop colitis, weeks, median (range)	5.1<br>(1.5-15.3)	N/A
Time from baseline to 1st time point, weeks, median (range)	5.4<br>(0.92-14.7)	6.7<br>(3.0-14.1)
Grade of colitis, n (%)		
G2	4 (21)	N/A
G3-4	15 (79)	
Objective response*, n (%)		
CR/PR/SD	9 (47)	11 (61)
PD	10 (53)	7 (39)
Other Toxicities, n (%)		
Rash	4 (21)	3 (17)
Vitiligo	3 (16)	2 (11)
Hypophysitis	3 (16)	0 (0)
Thyroid disorders	4 (21)	2 (11)
Pneunonitis	3 (16)	0 (0)
Hepatitis	4 (21)	0 (0)
AIDM	1 (5)	0 (0)

*** Abbreviations: CR, complete response; PR, partial response; SD, stable disease; PD, progression of disease**

Time points of PBMC collection:

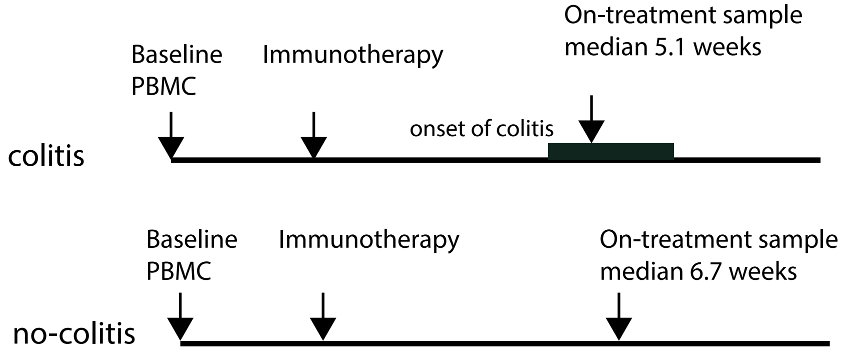

Suppl. Table 2: Metal-Conjugated antibodies used for CyTOF analysis

Label	Specificity	Clone	Manufacturer
**89**	CD8	RPA-T8	BioLegend
**104**	CD45	HI30	BioLegend
**108**	CD45	HI30	BioLegend
**113**	CD56	NCAM16.2	BD
**115**	Ki67	B56	BD
**141**	CD196 (CCR6)	11A9	BD
**142**	CD19	HIB19	BioLegend
**143**	CD117	104D2	BioLegend
**144**	TCRγδ	B1	BioLegend
**145**	PE	PE001	BioLegend
**146**	CD1c	L161	BioLegend
**147**	CD45RO	UCHL1	BioLegend
**148**	CD16	3G8	BD
**149**	CD45RA	HI100	BD
**150**	FITC	FIT-22	BioLegend
**151**	CD278 (ICOS)	DX29	BD
**152**	CD66b	G10F5	BD
**153**	CD68	KP1	BioLegend
**154**	CD3	UCHT1	BD
**155**	EOMES	WD1928	eBioscience
**156**	CD10	HI10a	BioLegend
**158**	CD33	WM53	BD
**159**	CD197 (CCR7)	150503	R&D Systems
**160**	CD14	M5E2	BD
**161**	CD274 (PD-L1)	29E.2A3	BioLegend
**162**	Foxp3	PCH101	eBioscience
**163**	CD183 (CXCR3)	G025H7	BioLegend
**164**	GATA3	L50-823	BD
**165**	Biotin	1D4-C5	BioLegend
**166**	CD185 (CXCR5)	RF8B2	BD
**167**	CD11c	Bu15	BioLegend
**168**	CD4	SK3	BioLegend
**169**	CD25	M-A251	BioLegend
**170**	CD152 (CTLAA-4)	14D3	eBioscience
**171**	CD24	ML5	BioLegend
**172**	Cy5	CY5-15	Sigma
**173**	CD141	AD5-14H12	Miltenyi Biotec
**174**	HLA DR	L243	BioLegend
**175**	CD279 (PD-1)	EH12.2H7	BioLegend
**176**	APC	APC003	BioLegend
**209**	Tbet	4B10	BD

Suppl.Table 3: Fluorophore-conjugated antibodies used for CyTOF analysis

mAb	Fluorophore	Clone	Manufacturer	
**CCR3**	BIOTIN	5B3	BioLegend	**Extracellular**
**CD161**	FITC	HP-3G10	BioLegend	**Extracellular**
**GrZB**	APC	GB11	BioLegend	**Intracellular**
**RORɣT**	Cy5	Q21-559	BD	**Intracellular**
**CD11b**	PE	ICRF44	BioLegend	**Extracellular**

Suppl. Table 4: Immunohistochemistry Antibody Panel

Order	Antibody	Source	Clone	Manufacturer	Dilution	Diluent	Opal	AR pH	Detection Kit
**Panel 1: T cell panel**									
**1**	CD3	Rabbit	MRQ-39	Cell Marque	1:2000	Antibody diluent /Block	690	9	Opal Polymer HRP Ms+Rb (Perkin Elmer)
**2**	FoxP3	Mouse	236A1E7	Abcam	1:2000	Antibody diluent /Block	620	9	Opal Polymer HRP Ms+Rb
**3**	T-bet	Rabbit	D6N8B	Cell Signalling	1;1000	Antibody diluent /Block	540	9	Mach 3 Rabbit HRP-polymer (Biocare Medical)
**4**	CD8	Rabbit	C8/144B	DAKO	1:1500	Antibody diluent /Block	520	9	Opal Polymer HRP Ms+Rb
**Panel 2: Cytotoxic T cell panel**									
**1**	GrZB	Mouse	GRB-7	DAKO	1:100	Antibody diluent /Block	520	9	Opal Polymer HRP Ms+Rb
**2**	CD103	Rabbit	EPR4166(2)	Abcam	1:1500	Antibody diluent /Block	620	9	Opal Polymer HRP Ms+Rb
**3**	CD8	Rabbit	C8/144B	DAKO	1:1500	Antibody diluent /Block	650	9	Opal Polymer HRP Ms+Rb
**4**	LAMP1	Rabbit	9091S	Cell Signalling	1:1000	Antibody diluent /Block	540	9	Opal Polymer HRP Ms+Rb
**5**	CD3	Rabbit	MRQ-39	Cell Marque	1:2000	Antibody diluent /Block	570	9	Opal Polymer HRP Ms+Rb
**6**	Ki67	Rabbit	D2H10	Cell Signalling	1:2000	Da Vinci Green (Biocare Medical)	690	9	Opal Polymer HRP Ms+Rb
**Panel 3: Myeloid panel**									
**1**	CD14	Rabbit	EPR16784	Cell Marque	1:100	Antibody diluent /Block	540	6	Mach 3 Rabbit HRP-polymer
**2**	MPO	Rabbit	A039829-2 Polyclonal	DAKO	1:2500	Antibody diluent /Block	570	9	Mach 3 Rabbit HRP-polymer
**3**	FXIIIA	Sheep	Polyclonal	Affinity Biological	1:5000	Antibody diluent /Block	520	9	1:5000 Sheep secondary in TBST
**4.**	CD56	Rabbit	MRQ-42	Cell Marque	1:500	Antibody diluent /Block	620	9	Opal Polymer HRP Ms+Rb

**5.**	CD16a	Rabbit	EPR20627	Abcam	1:800	Antibody diluent /Block	690	9	Opal Polymer HRP Ms+Rb

Supplementary Table 5: Cell phenotyping by multiplex immunohistochemistry

**Cell type**	**Phenotype**
**T cell panel**	
**T cells**	DAPI+CD3+
**CD8 T cells**	DAPI+CD3+CD8+
**CD4 T cells**	DAPI+CD3+CD8-
**Tregs**	DAPI+CD8-FoxP3+
**Th1 cells**	DAPI+CD3+CD8-Tbet+
**Activated CD8 T cells**	DAPI+CD3+CD8+Tbet+
**Myeloid panel**	
**Natural Killer (NK cells)**	DAPI+CD56+
**Fibroblast**	DAPI+CD14-CD16-FXIIIa+
**Granulocyte**	DAPI+MPO+CD16-
**Neutrophil**	DAPI+ MPO+CD16+
**Eosinophil**	DAPI+MPO+CD16-
**Classical monocytes**	DAPI+CD14+CD16-
**Non- classical monocytes**	DAPI+CD14-CD16+
**Intermediated monocytes**	DAPI+CD14+CD16+
**Cytotoxic T cell panel**	
**CD8+Ki67+**	DAPI+CD3+CD8+Ki67+
**CD8-Ki67+**	DAPI+CD3+CD8-Ki67+
**Tissue resident memory T cells**	DAPI+CD3+CD8+CD103+
**Proliferating Tissue resident memory T cells**	DAPi+CD3+CD8+CD103+Ki67+
**Non-tissue resident memory T cells**	DAPI+CD3+CD8+CD103-
**CD8+GrZB+**	DAPI+CD3+CD8+GrZB+
**CD8+LAMP1+**	DAPI+CD3+CD8+LAMP1+
**CD8+GrZB+LAMP1+Ki67+**	DAPI+CD3+ CD8+GrZB+LAMP1+Ki67+
